# A Review of *Ganoderma* Triterpenoids and Their Bioactivities

**DOI:** 10.3390/biom13010024

**Published:** 2022-12-22

**Authors:** Mahesh C. A. Galappaththi, Nimesha M. Patabendige, Bhagya M. Premarathne, Kalani K. Hapuarachchi, Saowaluck Tibpromma, Dong-Qin Dai, Nakarin Suwannarach, Sylvie Rapior, Samantha C. Karunarathna

**Affiliations:** 1 Center for Yunnan Plateau Biological Resources Protection and Utilization, College of Biological Resource and Food Engineering, Qujing Normal University, Qujing 655011, China; 2 Postgraduate Institute of Science (PGIS), University of Peradeniya, Peradeniya 20400, Sri Lanka; 3 Institute of Chemistry, Chinese Academy of Sciences, Beijing 100190, China; 4 National Institute of Fundamental Studies (NIFS), Hanthana, Kandy 20000, Sri Lanka; 5 The Engineering Research Center of Southwest Bio-Pharmaceutical Resource Ministry of Education, Guizhou University, Guiyang 550025, China; 6 Research Center of Microbial Diversity and Sustainable Utilization, Faculty of Science, Chiang Mai University, Chiang Mai 50200, Thailand; 7 Laboratory of Botany, Phytochemistry and Mycology, Faculty of Pharmacy, Univ Montpellier, 15 Avenue Charles Flahault, CS 14491, CEDEX 5, 34093 Montpellier, France; 8 CEFE, Univ Montpellier, CNRS, EPHE, IRD, Natural Substances and Chemical Mediation Team, 15 Avenue Charles Flahault, CS 14491, CEDEX 5, 34093 Montpellier, France

**Keywords:** bioactivity, bioactive molecules, drug, Lingzhi, Reishi, triterpenes

## Abstract

For centuries, *Ganoderma* has been used as a traditional medicine in Asian countries to prevent and treat various diseases. Numerous publications are stating that *Ganoderma* species have a variety of beneficial medicinal properties, and investigations on different metabolic regulations of *Ganoderma* species, extracts or isolated compounds have been performed both in vitro and in vivo. However, it has frequently been questioned whether *Ganoderma* is simply a dietary supplement for health or just a useful “medication” for restorative purposes. More than 600 chemical compounds including alkaloids, meroterpenoids, nucleobases, nucleosides, polysaccharides, proteins, steroids and triterpenes were extracted and identified from *Ganoderma*, with triterpenes serving as the primary components. In recent years, *Ganoderma* triterpenes and other small molecular constituents have aroused the interest of chemists and pharmacologists. Meanwhile, considering the significance of the triterpene constituents in the development of new drugs, this review describes 495 compounds from 25 *Ganoderma* species published between 1984 and 2022, commenting on their source, biosynthetic pathway, identification, biological activities and biosynthesis, together with applications of advanced analytical techniques to the characterization of *Ganoderma* triterpenoids.

## 1. Introduction

*Ganoderma* P. Karst. 1881 [1] is an old polypore genus typified by *Ganoderma lucidum* (Curtis) P. Karst. belonging to the family Ganodermataceae (basidiomycete) [2], which has been synonymized with the family Polyporaceae [3], with the members normally growing on woody plants and logs [2,4,5]. The genus was originally reported in the UK [6] and is considered to have a worldwide distribution [7,8,9,10,11,12,13,14,15,16,17,18]. *Ganoderma* species are used for medicinal purposes in China [19,20,21,22], Japan, South Korea [22] and the French West Indies [12]. The common names for *Ganoderma* include Lingzhi, Munnertake, Sachitake, Reishi, and Youngzhi. *Ganoderma* was first recorded in Shennong’s Classic of Materia Medica and classified as an upper-grade medicine in medical books [23]. Index Fungorum (2022) (http://www.indexfungorum.org/, accessed on 6 September 2022) lists 488 records of *Ganoderma* while MycoBank lists 529 records [18]. Approximately 80 species of *Ganoderma* are recorded in Chinese Fungi [24], of which *G. lucidum* and *G. sinense* are described as medicinally beneficial macrofungi in Chinese medicine [25]. However, other species, such as *G. capense*, *G. cochlear* and *G. tsugae* also play an important role in traditional folk medicine. In addition, pharmacological studies have also used the extract and chemical constituents of other *Ganoderma* species [22,26,27,28,29,30].

The chemical constituents and the biological activities of 25 species of *Ganoderma*, namely *G. amboinense, G. annulare, G. applanatum* (synonym *G. lipsiense*), *G. australe, G. boninense, G. capense, G. carnosum, G. casuarinicola, G. cochlear, G. colossus, G. concinnum, G. ellipsoideum, G. fornicatum, G. hainanense, G. lucidum, G. mastoporum, G. neo-japonicum, G. orbiforme, G. pfeifferi, G. resinaceum, G. sinense, G. theaecola* (as *theaecolum*), *G. tropicum, G. tsugae* and *G. weberianum* were studied and reported in this review. Several chemical constituents such as ganoderic acids, lucidenic acids, 12-hydroxyganoderic acid, ganorbiformin, lucidimines [31] and other compounds have been reported from various *Ganoderma* species during recent decades [29,32,33,34,35].

Triterpenes, polysaccharides and peptidoglycans are one of the major types of bioactive substances [36,37] responsible for the various biological activities of several species in *Ganoderma* (e.g., *Ganoderma lucidum*). Triterpenoids and ganoderic acids that play a critical role in pharmacological activities are also present in *G. applanatum* and *G. orbiforme* [33,36,38,39,40,41,42]. Besides *G. applanatum* [43,44], *G. colossus* [45] and *G. pfeifferi* [46]*, G. resinaceum* [45] have also been identified as potential *Ganoderma* natural antioxidants.

*G. amboinense* [47], *G. annulare* [48], *G. applanatum* [49,50,51], *G. boninense* [52], *G. calidophilum* [53], *G. capense* [54], *G. carnosum* [55], *G. cochlear* [56,57,58], *G. colossum* [59], *G. concinnum*, *G. neo-japonicum* [60], *G. fornicatum* [61], *G. hainanense* [62], *G. leucocontextum* [63], *G. lucidum* [64,65,66,67], *G. mastoporum* [68], *G. mbrekobenum* [69], *G. resinaceum* [70], *G. sinense* [71,72,73], *G. theaecola* (as *theaecolum*) [74], *G. tropicum* [75], *G. tsugae* [76], *G. weberianum* [77] were reported to contain bioactive compounds of great interest.

The members of the genus *Ganoderma* are rich in novel “mycochemicals”, including polysaccharides [78], steroids, fatty acids, phenolic compounds, vitamins, amino acids and triterpenoids [69,79,80,81]. *Ganoderma* polysaccharides are a hot topic in the medicinal mushroom research field [78,82,83,84]. Although polysaccharides are found to be one of the main bioactive constituents, their high molecular weight and complex structure limit their use in the drug market [85]. The *Ganoderma* polysaccharides are well known for their diverse bioactivities such as antitumor, immunomodulatory, anti-hypertensive, antidyslipidemic and hepatoprotective activities. *Ganoderma* polysaccharides have been developed into medicines, dietary supplements, healthy foods, treat and prevent diseases, and are continuing to serve as important leads in modern drug discovery [78,86,87,88]. 

Previous reviews were mainly focused on the anticancer properties [81,89,90,91,92,93,94,95,96], hepatoprotective activities [97], antimicrobial activities [42] and mechanisms of *Ganoderma* triterpenoids [35,98,99,100]. 

Herein, we review the structures, bioactivities, and biosynthesis pathways of the small molecular constituents of triterpenoids from 25 species of *Ganoderma* to lay a foundation for further research on the development of new medicines. 

### Taxonomic Studies of Ganoderma

We review 25 *Ganoderma* species described between 1984 and 2022 in this article. The word *Ganoderma* is derived from the Greek words “Gano”, meaning “shiny”, and “derma”, meaning “skin” [22,101]. Originating from the tropics and recently extending its range into temperate zones [102], *Ganoderma* is an old genus with a taxonomic history dating back to 1881, and the Finnish mycologist P. A. Karsten erected the genus to place a single species, *Polyporus lucidus* [=*Ganoderma lucidum* (Curtis: Fr.) P. Karst.] [1,103]. *Ganoderma lucidum* is the type species of the genus *Ganoderma*, which was originally described as from the UK and later found to have a worldwide distribution [6]. A number of *Ganoderma* species morphologically closely related and belonging to *G. lucidum* complex viz. *G. multipileum* Ding Hou 1950 [104], *G. sichuanense* J.D. Zhao & X.Q. Zhang 1983 [105] and *G. lingzhi* Sheng H. [106,107] from China, *G. resinaceum* Boud. 1889 [108] from Europe, and *G. oregonense* Murrill 1908, *G. sessile* Murrill 1902, *G. tsugae* Murrill 1902 and *G. zonatum* Murrill 1902 [109,110] from the USA, have been described as from all over the world, and are mainly characterized by laccate pileus. Zhou et al. [111] considered 32 collections belonging to the *G. lucidum* complex from Asia, Europe and North America in terms of their morphology and phylogeny as derived from analyses of four protein-coding genes viz. Internal transcribed spacer (ITS), translational elongation factor 1-*α* (*tef1-α*) and retinol-binding protein 1 and 2 (*rpb1*, *rpb2*). Different molecular techniques have been used to study the genetic diversity in *Ganoderma*, such as amplified fragment length polymorphism, isozyme analysis, inter simple sequence repeat, random amplified polymorphic DNA, single-nucleotide polymorphism, restricted fragment length polymorphisms, sequence-related amplified polymorphism, single-strand conformational polymorphism and sequence characterized amplified region [112]. These different molecular identifications used in different taxonomic classifications of *Ganoderma* have caused great improvements and provide information for the further research of *Ganoderma* [18,102,113].

*Ganoderma* has long been treated as one of the most important medicinal fungi worldwide [39], and laccate species of *Ganoderma* have been considered for centuries [114], i.e., *G. lucidum* complex have been used as medicinal mushrooms in traditional Chinese medicine [115]. Anyhow, the species concept of the *G. lucidum* complex lacks agreement in morphology, and the taxonomy of this species complex is thus problematic, and this ultimately limits both further research on this complex and their medicinal usefulness. For example, the widely used medicinal species in biochemical and pharmaceutical studies have been assumed to be *G. lucidum*, but evidence has emerged that this medicinal species is, in fact, a different species [116] and was described as *G. lingzhi* [106].

The taxonomic position of the *G. lucidum* complex has long been subjected to debate [9,18,102,113,117] and different opinions have been expressed regarding the members and their validity in the complex. Haddow [118] treated *G. sessile* as a synonym of *G. resinaceum*, while Overholts [119] pointed out that *G. lucidum* should be the correct name of the specimens classified as *G. sessile. Ganoderma lucidum* has also been considered as the correct name over its later synonym *G. tsugae* [118,120]. However, with the support of mating tests, Nobles [121] pointed out that the specimens classified as *G. lucidum* in the USA represented *G. sessile*. All names of the *G. lucidum* complex in East Africa were parsimoniously treated as the ‘‘*G. lucidum* group’’, because of the lack of morpho-taxonomic solution to the problem in this complex [7]. Asian specimens classified as *G. lucidum* were divided into two clades; both were separated from European *G. lucidum* [122], with one clade being composed of tropical collections represented by *G. multipileum*, while the other clade was unknown [122], yet later recognized as *G. sichuanense* [123]. It was found that the holotype of *G. sichuanense* was not conspecific with the unknown clade, and the unknown clade was identified as a new species, *G. lingzhi*, which also is the most widely cultivated species in China [106]. Meanwhile, the distribution of genuine *G. lucidum* in China was also confirmed [106,124]. The taxonomy of the *G. lucidum* complex remains problematic even after several decades of debates [111]. Most of the studies previously conducted were focused on the species in a continent or specific region [106,123], or the phylogeny was described with low resolution to certain clades [116,124]. On the other hand, a strong phylogeny together with species originally described as from the USA is greatly needed, as most of these species are old and never referred to in any phylogenetic analyses.

## 2. Triterpenoids

### 2.1. Biosynthesis of Triterpenoids

Triterpenoids belong to a large and structurally diverse class of natural products [125]. Basidiomycetes are considered a main source for triterpenoids. Compared to plants, very few types of triterpenoid skeletons have been reported in basidiomycetes, thus more research is needed [126]. Triterpenoids extracted from *Ganoderma* spp. are named as *Ganoderma* triterpenoids (GTs) [125]. 

**Figure 1:**
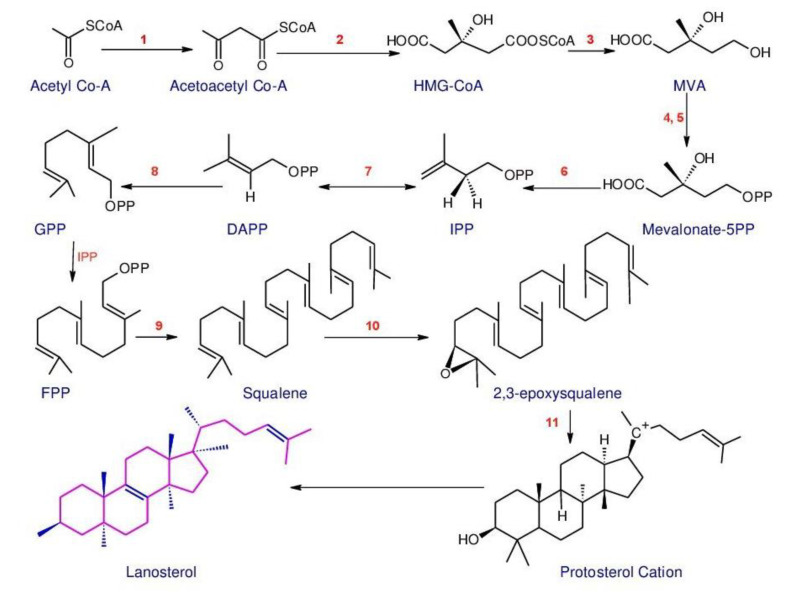
Outline of the MVA and lanostane-type triterpenoids biosynthesis. Enzymes involved in the pathway are: 1. Acetyl-CoA acetyltransferase, AACT; 2. 3-Hydroxy-3-methylglutaryl-CoA synthase, HMGS; 3. 3-Hydroxy-3-methylglutaryl-CoA reductase, HMGR; 4. Mevalonate kinase, MK; 5. Phosphomevalonate kinase, MPK; 6. Phosphomevalonate decarboxylase, MVD; 7. Isopentenyldiphosphate isomerase, IDI; 8. Farnesyl diphosphate synthase, FPPs; 9. Squalene synthase, SQS; 10. Squalene monooxygenase, SE; 11. 2,3-Oxidosqualene-lanosterol cyclase, OSC (The structures were redrawn in ACD/ChemSketch: Freeware: 2012).

*Ganoderma* triterpenoids (GTs) isolated from the fruiting bodies, cultured mycelia and basidiospores of *Ganoderma* [127,128,129] belong to the lanostane-type triterpenoids and are one of the major chemical constituents in *Ganoderma*. Studies have confirmed that GTs are biosynthesized using isoprenoid pathways [130,131]. This pathway was considered to start from acetyl-coenzyme A and termed as a Mevalonate pathway (MVA) [132]. The MVA pathway (Figure 1) is one of the important metabolic pathways that can be divided into four main processes: conversion, construction, condensation, and postmodification [35]. The initial step is the transformation of acetyl-coenzyme A to isopentenyl pyrophosphate (IPP). Then the activities of different prenyltransferases produce farnesyl pyrophosphate (FPP), geranyl pyrophosphate (GPP) and geranylgeranyl pyrophosphate (GGPP), which are higher-order terpenoid building blocks from this IPP precursor. Then, these junction mediators can self-condense, and are also used in alkylation processes to produce prenyl side chains (for a range of nonterpenoids) or create a ring (to build the basic skeletons of triterpenoids). Ultimately, oxidation and reduction reactions, conjugation, isomerization or other secondary reactions magnify the distinctive characteristics of the triterpenoids [35,133,134].

The relationship between the content of *Ganoderma* triterpenoids and the expression levels of major genes has been evaluated in several studies. These kinds of studies may provide valuable data for studying the role of key genes and, finally, for raising the production of triterpenoids. According to Wang et al. [126], the most comprehensive studies on *Ganoderma* triterpenoids were concentrated on *G. lucidum*. A considerable number of essential enzyme genes are involved in the production of *G. lucidum* triterpenoids during the biosynthesis. Liu et al. [135] inspected the *G. lucidum* genes in the “terpenoid backbone biosynthesis (map00900)” pathway and discovered that the genes are solely spread in the MVA pathway, not the MEP/DOXP (methylerythritol 4-phosphate/deoxyxylulose 5-phosphate) pathway. Meanwhile, several genes in this pathway have been cloned in *G. lucidum*, including 3-hydroxy-3-methylglutaryl-CoA reductase (HMGR) [136], Farnesyl diphosphate synthase (FPPs) [137], squalene synthase (SQS) [138], and lanosterol synthase (also namely 2,3-oxidosqualene lanosterol cyclase, OSC) [135]. These observations demonstrated that triterpenoid backbone biosynthesis could only be accomplished via the MVA pathway at the genome level in fungi.

Scientists have performed a large number of studies to find out key enzyme genes involved in increasing the yield of ganoderic acid (GA). Ye et al. [139] examined the effect of salicylic acid (SA) and calcium ions on the biosynthesis of triterpenoids in *G. lucidum* through spraying during the fruiting stage. Calcium ions had no effect on the production of triterpenoids because the six key triterpenoid biosynthetic genes did not respond. However, SA increased triterpenoid content by 23.32% compared to the control, and the combined induction of both increased triterpenoid content by 13.61% compared to the control since in the case of SA and the combined induction of both on the six-triterpenoid biosynthetic genes were up-regulated [139]. Fei et al. [140] have utilized the homologous farnesyl diphosphate synthase gene to overexpress GA (which actually increases the generation of GA) to determine the function of MVD in the biosynthesis of GA. However, it also increases expression of squalene synthase (SQS) and lanosterol synthase (LS). 

According to the results of Shi et al. [141], it was revealed that MVD plays a key role in the biosynthesis of GA. The SE gene was cloned from *G. lucidum* and overexpressed to examine the impact on the biosynthetic pathway of GA. The results of this study indicated that the SE gene promotes the biosynthesis of GA. In addition, SE and HMGR genes were simultaneously expressed during this study, with the co-expressed strain having a higher acid content than the single expressed strain, which demonstrated that the co-expression of the two genes stimulated biosynthesis of GA [113,142,143]. LS is a key enzyme of the MVA synthesis pathway and is at the second branch point, [113,126,142,143] whilst it was revealed that the overexpression of LS increases the content of GA [113,142,143]. As a summary, the fundamental enzyme gene in the biosynthesis pathway of GA is intensely involved in the amount of GA.

### 2.2. Structures and Bioactivities of Triterpenoids

Triterpenes are a major class of widely dispersed secondary metabolites in nature [144]. Triterpenoids structures with a carbon skeleton are considered to be derived from the acyclic precursor squalene [145]. More than 30,000 structures of triterpenes [146] such as dammarane, lanostane, lupine, oleanane and ursane types have been isolated and identified [147].

**Figure 2:**
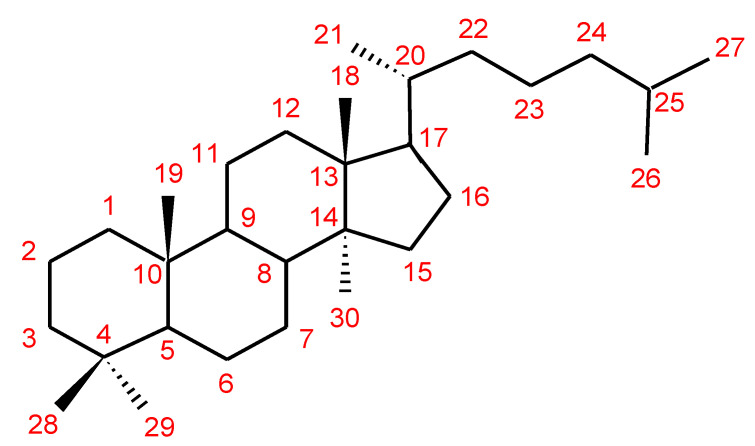
Chemical structure of lanostane triterpene.

The structures of triterpenoids isolated from *Ganoderma* spp. are complicated. These compounds consist with lanostane carbon skeleton and pentacyclic triterpenoids (Figure 2). According to the number of carbon atoms in their skeleton, GTs can be divided into three types viz. C30, C27 and C24 [148]. On the basis of the substituent groups, they are classified into different groups such as triterpenoid acids, triterpenoid alcohols and triterpenoid lactones [148].

With the development of separation techniques and extraction methods, structurally diverse triterpenoids were isolated [149,150], which were formed through oxidation, reduction, cleavage, rearrangement and cyclization within MVA pathway [151]. It is known that the bioactivity of GTs has long been the research focus and that new changes of structures can affect their bioactivities [29,152]. Thus, we summarize the triterpenoids of 25 species of *Ganoderma* and classify them into seven types on the basis of the number and type of skeleton carbons (i.e., C31, C30, C29, C27, C25, C24, and rearranged novel skeleton).

#### 2.2.1. C30 Triterpenoids

The majority of GTs was C30 triterpenoids on the basis of the biosynthetic pathway of GTs. Because of the oxidation and reduction processes, their structures should be divided into six groups: 8,9-ene, 8,9-dihydro, 8,9-epoxy, 7(8),9(11)-diene, triterpenoid saponins and rearranged novel skeleton triterpenoids.

##### 8,9-ene-triterpenoids

This type of GT is the lanostane-type triterpenoid with a double bond between C-8 and C-9. In addition, C-3, C-7, C-11 and C-15 were generally substituted by the hydroxyl or carbonyl groups. The minority of compounds possessed hydroxyl or carbonyl groups at C-12. Different oxidation and reduction can happen in the side chain. Except the above structural features, compounds ganodermacetal (**44**) and methyl ganoderate A acetonide (**45**) possessed an uncommon acetonide unit. Compound **44** was isolated from the basidiomycete *G. amboinense*, and was a natural product, but not an artefact, which resulted from the acetalization of native ganoderic acid C (**3**) and with acetone not being used during the isolation procedure [153]. However, compound **45** isolated from the fruiting bodies of *G. lucidum* was reported to be most likely not of natural origin due to the utilization of acetone during the isolation procedure [154]. 

Several studies on the bioactivity of triterpenoids showed that ganoderic acids had significant biological activities [107,155,156,157,158,159] (Table 1). Ganoderic acid DM (**50**) displayed stronger 5*α*-reductase inhibitory activity (IC_50_ value of 10.6 μM) than the positive control (*α*-linolenic acid, 116 μM). Meanwhile, compared to its methyl derivative, the inhibitory activities of 5*α*-reductase at 20 μM were 55**%** and 3**%** for **50** and its derivative, suggesting that the carboxyl group of the side chain for **50** is essential to elicit the inhibitory activity [155]. Liu et al. [156] evaluated the structure –activity relationship for inhibition of 5*α*-reductase using ganoderic acid A (**1**)**,** B (**2**)**,** C (**3**)**,** D (**19**)**,** I (**5**) and DM (**50**)**.** The results showed that the presence of the carbonyl group at C-3, and of the *α*,*β*-unsaturated carbonyl group at C-26, was characteristic of almost all inhibitors. 

**Figure 3:**
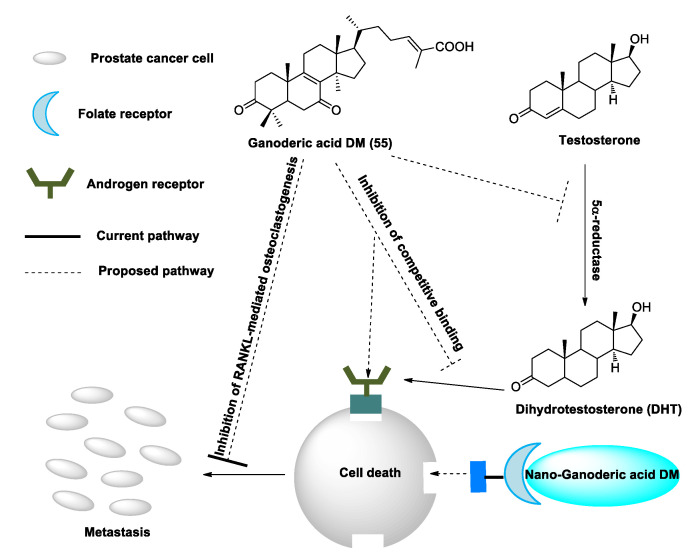
Suggested mechanisms by which ganoderic acid DM (GA-DM) may inhibit prostate cancer cell proliferation and metastasis.

Except for the aforementioned bioactivity, ganoderic acid DM (**50**) was also a selective potent osteoclast genesis inhibitor [157,158]. Miyamoto et al. [159] found that ganoderic acid DM (**50**) can suppress the expression of c-Fos and the nuclear factor of activated T cells c1 (NFATc1). This suppression leads to the inhibition of the dendritic cell-specific transmembrane protein (DC-STAMP) expression and reduces osteoclast fusion. The study of Liu et al. [157,158] also displayed that this compound can be used in therapeutics for prostate cancer by inhibiting the cancer cell proliferation and bone metastases by impeding the osteoclast differentiation. Meanwhile, Johnson et al. [160] indicated the possible mechanism by which ganoderic acid DM (**50**) induces cytotoxicity in both androgen-dependent and independent prostate cancer cells (Figure 3). Compound **50** can also induce DNA damage, G1 cell cycle arrest and apoptosis in human breast cancer cells [107]. Meanwhile, tubulin was identified as the target protein of ganoderic acid DM (**50**)**,** which can explain and clarify the universal mechanism of its medicinal efficacy [161].

Ganoderic acid Df (**22**) is a ganoderma acid having a 23-oxo-24-en-26 oic acid side chain, different from ganoderic acid DM (**50**) which has a 24-en-26 oic acid side chain. Compound **22** exhibited potent human aldose reductase inhibitory activity with an IC_50_ of 22.8 μM in vitro, with the carboxyl group of this compound’s side chain being essential for eliciting inhibitory activity because its methyl ester is much less active [162]. Similarly, Fatmawati et al. [163] analyzed the structure–activity relationships of ganoderma acids (ganoderic acids A, B, C, D, H, J, K, Df, ganoderenic acids A and D, as well as methyl ganoderate A, methyl ganoderenate A, and ganolucidic acid B) from *G. lucidum* as aldose reductase inhibitors. The results revealed that the OH substituent at C-11 is a valuable feature and that the carboxyl group in the side chain is essential for the recognition of aldose reductase inhibitory activity. Moreover, double bond moiety at C-20 and C-22 in the side chain contributes to improving aldose reductase inhibitory activity. All OH substituents at C-3, C-7 and C-15 are valuable for potent aldose reductase inhibition. Fatmawati et al. [164] explained the structural requirements for *α*-glucosidase inhibition. The structure–activity relationships of ganoderma acids revealed the same results as the above research.

**Figure 4:**
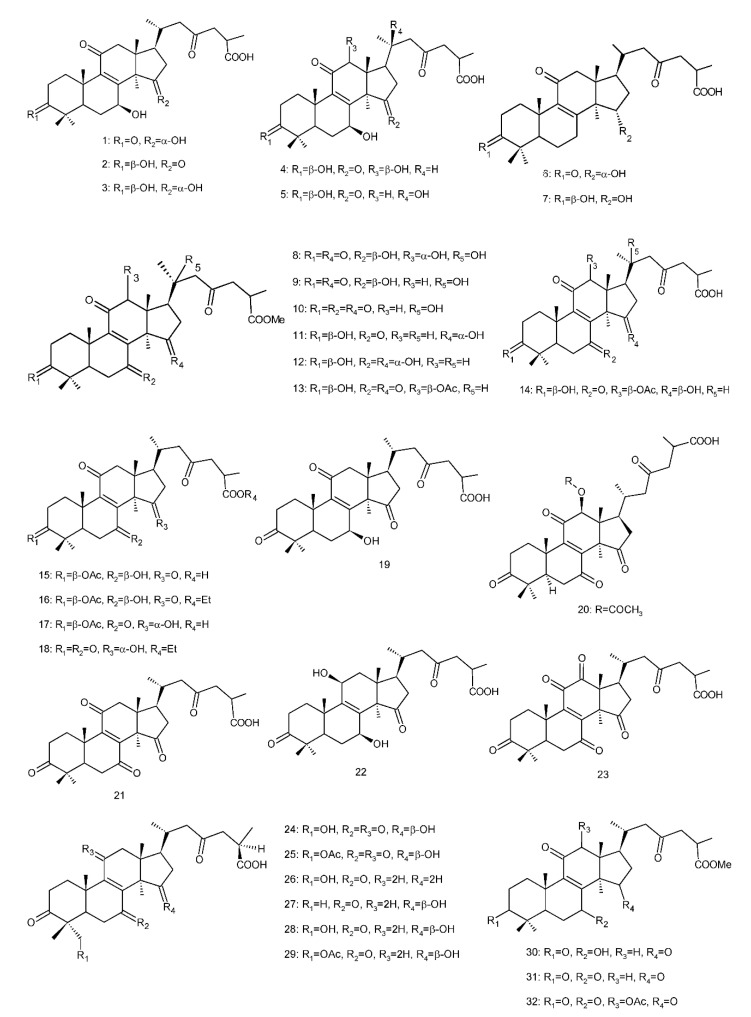

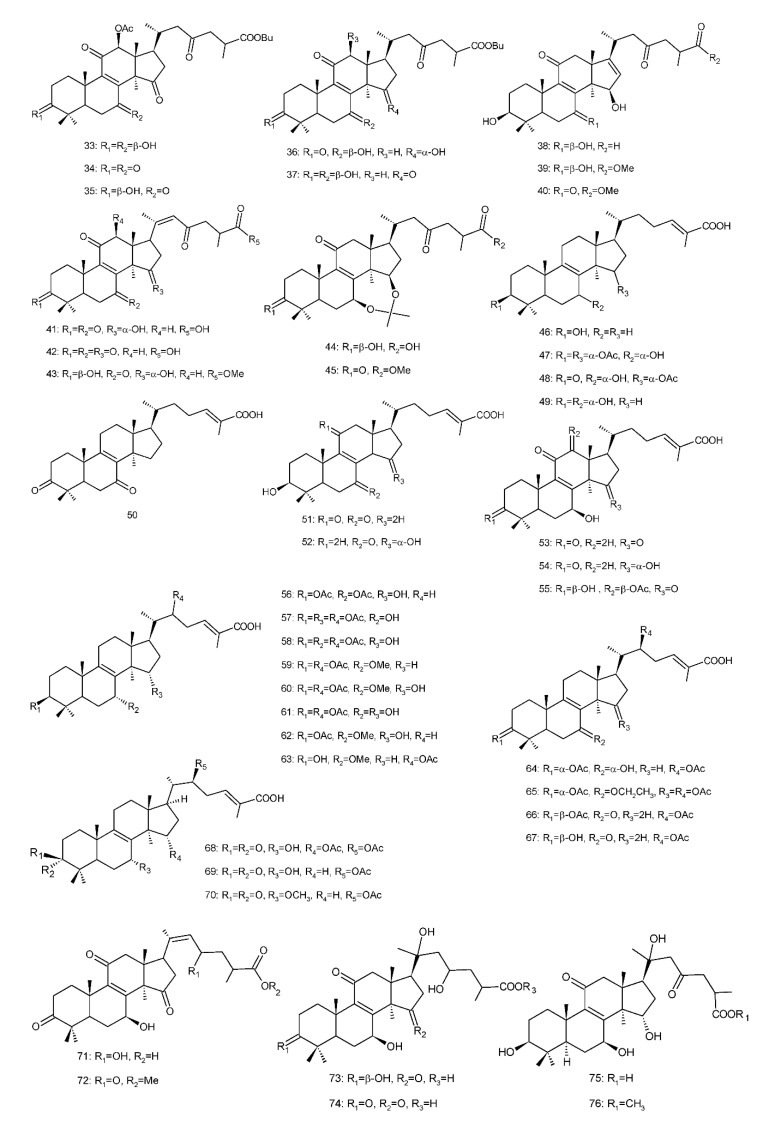

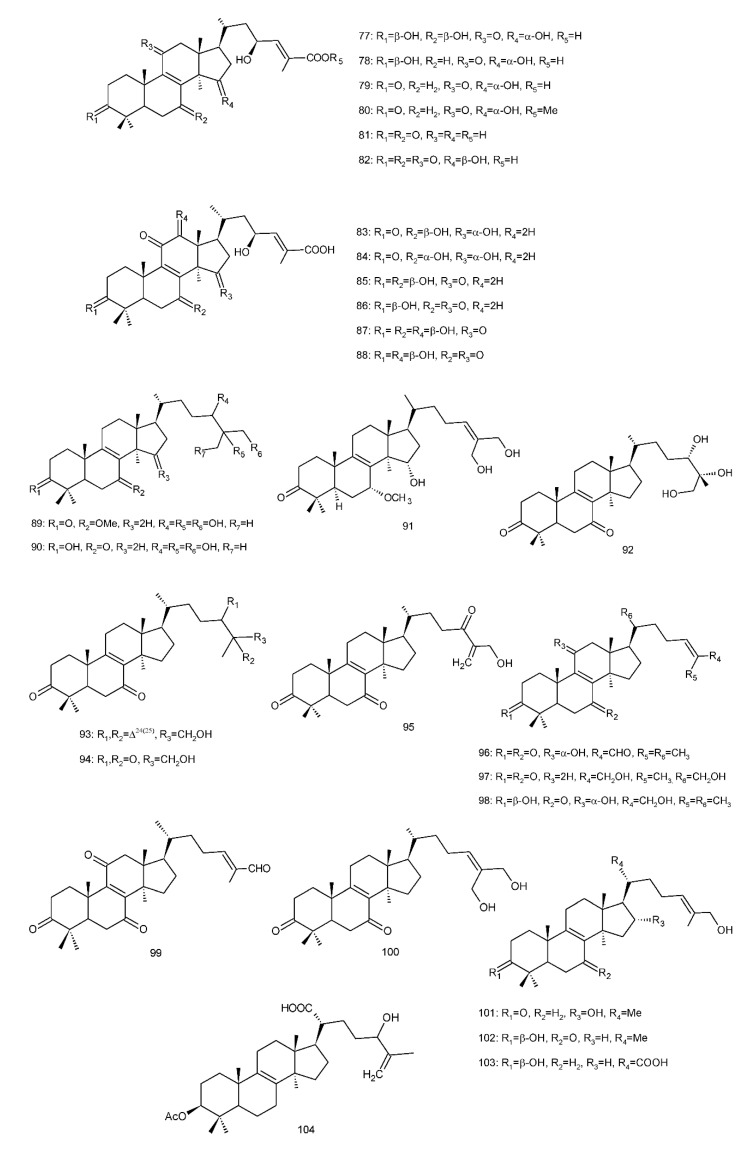

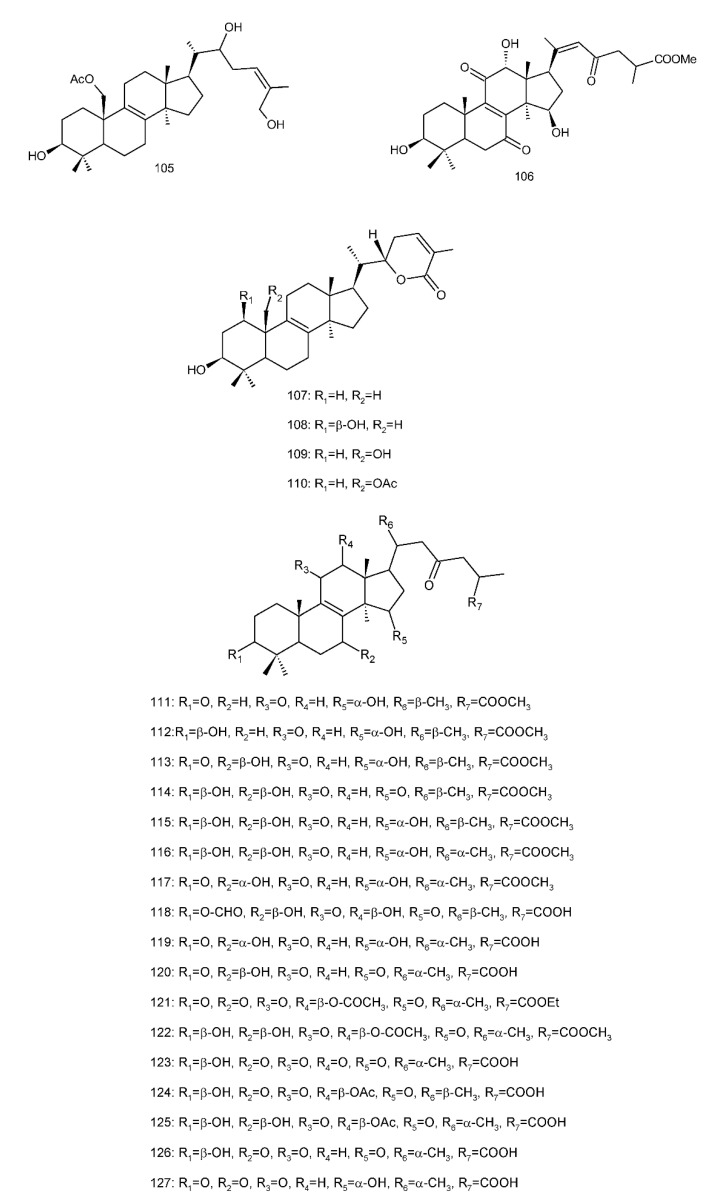

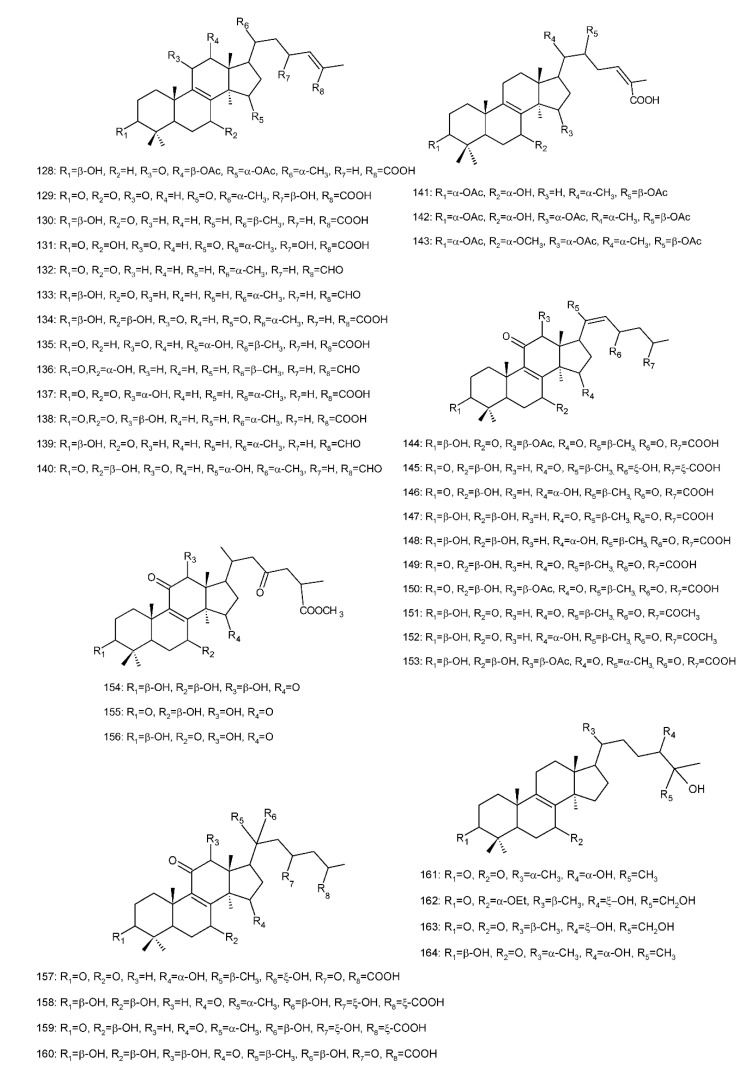

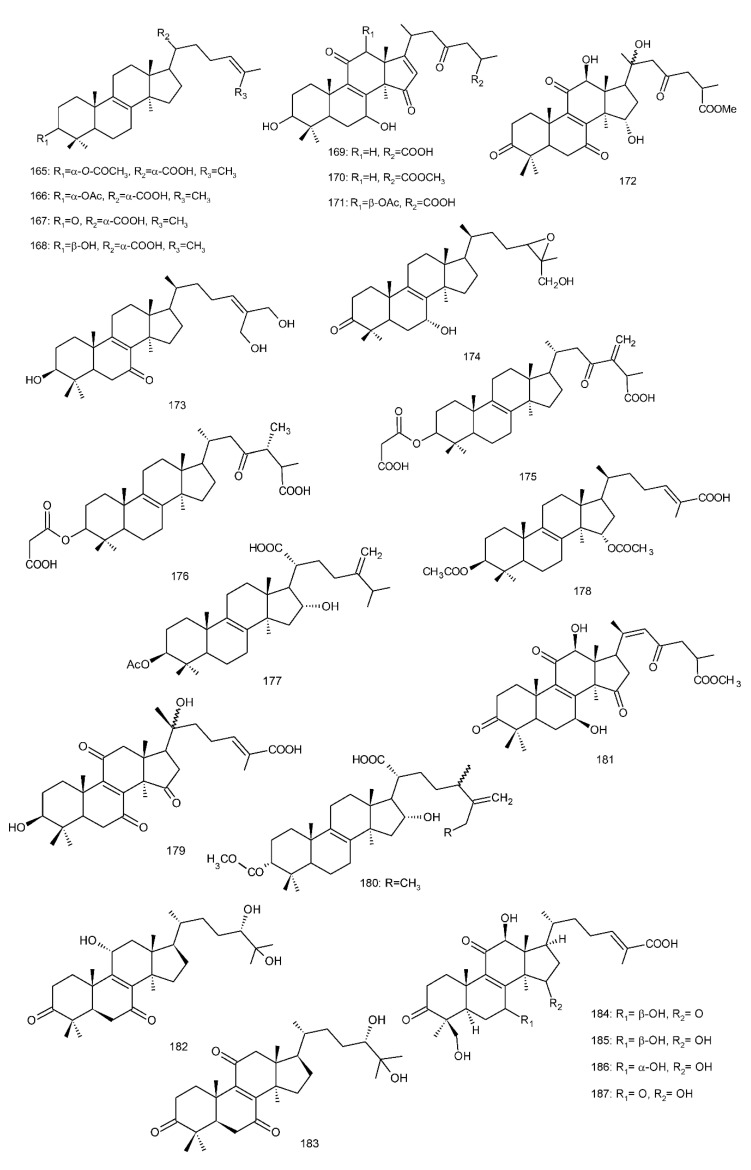

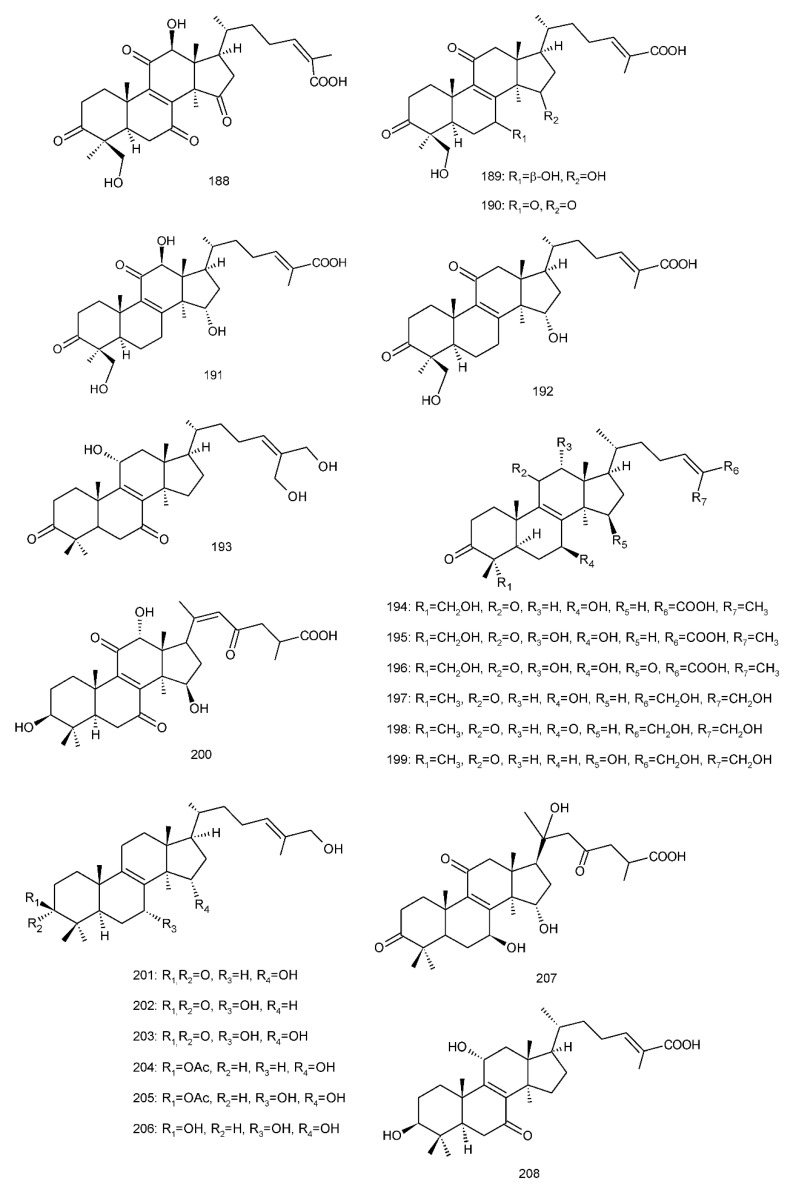

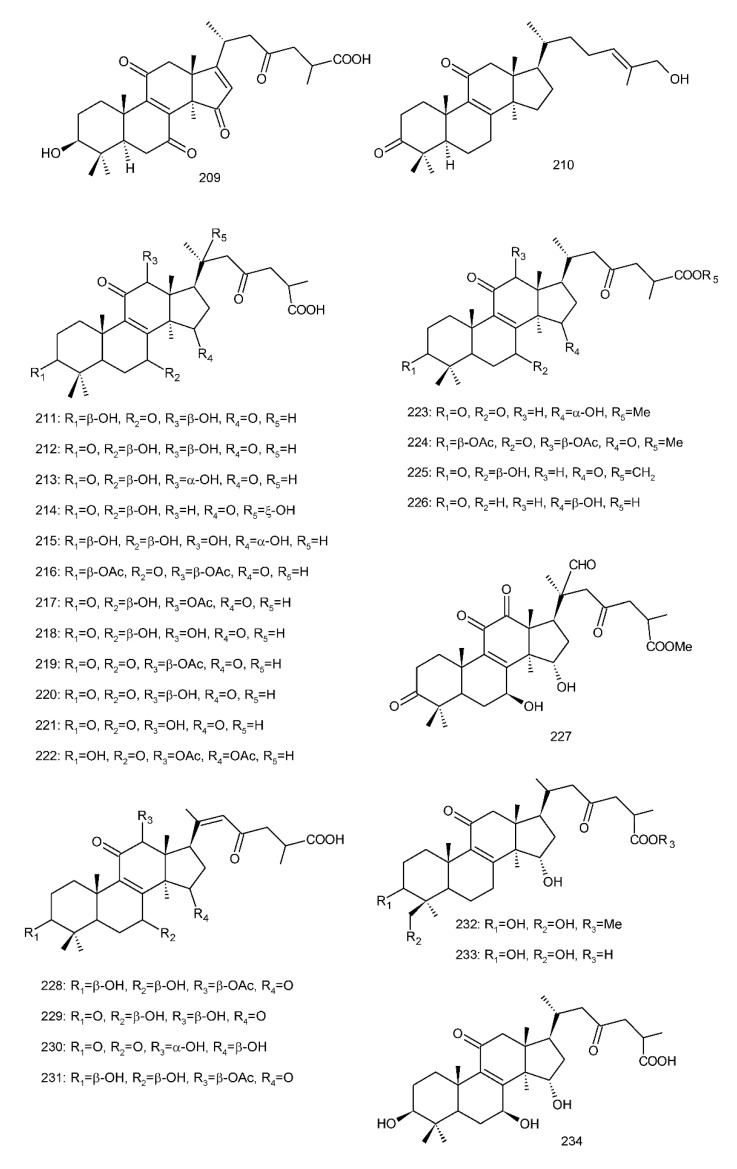

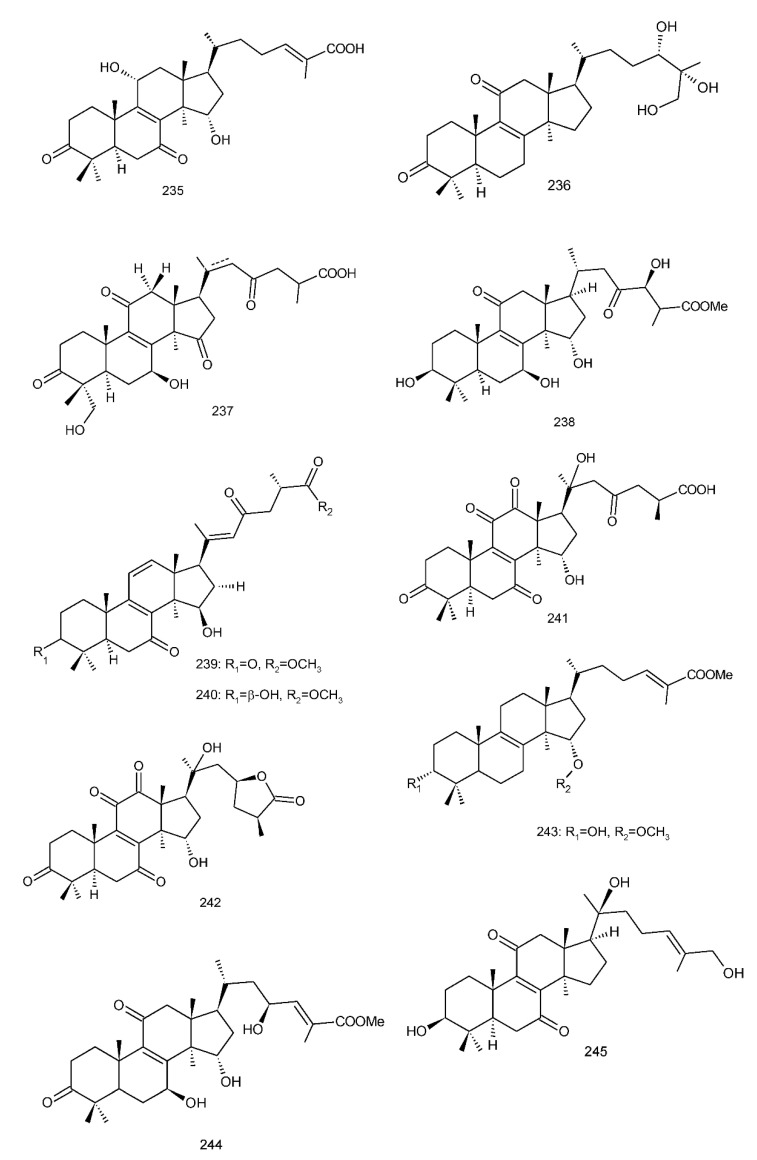
Chemical structures of several 8,9-double-bond triterpenoids (1–245).

In all, C30 ganoderma acids showed potent metabolic enzyme inhibitory activities, and the carboxyl group in the side chain was the key factor (Figure 4). 

**Table 1:** 8,9-double-bond triterpenoids and bioactivities from *Ganoderma*.

No.	Trivial Names	Bioactivities (IC_50_/MIC or ED_50_)	Sources *Ganoderma* Species	References
**1.**	Ganoderic acid A	Promising anticancer agent (via potent inhibitory effect on JAK/STAT3 pathway)	*G. lucidum*, *G. tsugae*	[165,166,167]
**2.**	Ganoderic acid B	Moderately active inhibitor against HIV-1 PR (0.17 mM)	*G. lucidum*, *G. tsugae*	[165,166,168]
**3.**	Ganoderic acid C	Suppressed LPS-induced TNF-α (IC_50_ = 24.5 µg/mL) production through down-regulating MAPK, NF-kappa B and AP-1 signaling pathways in macrophages	*G. lucidum*, *G. tsugae*	[165,169]
**4.**	Ganoderic acid G	Antinociceptive effect	*G. lucidum*	[170,171]
**5.**	Ganoderic acid I	Cytotoxicity against Hep G2 cells (IC_50_ = 0.26 mg/mL), HeLa cells (IC_50_ = 0.33 mg/mL), Caco-2 cells (IC_50_ = 0.39 mg/mL)	*G. lucidum*	[170,172]
**6.**	Ganolucidic acid A	-	*G. lucidum*	[173]
**7.**	Ganolucidic acid B	-	*G. lucidum*	[174]
**8.**	Methyl ganoderate M	-	*G. lucidum*	[175]
**9.**	Methyl ganoderate N	-	*G. lucidum*	[175]
**10.**	Methyl ganoderate O	-	*G. lucidum*	[175]
**11.**	Methyl ganoderate K	-	*G. lucidum*	[175]
**12.**	Compound B9	-	*G. lucidum*	[175]
**13.**	Methyl ganoderate H	-	*G. lucidum*	[175]
**14.**	Ganoderic acid *α*	Anti-HIV protease (0.19 mM)	*G. lucidum*	[168]
**15.**	3-O-acetylganoderic acid B	-	*G. lucidum*	[176]
**16.**	Ethyl 3-O-acetylganoderate B	-	*G. lucidum*	[176]
**17.**	3-O-Acetylganoderic acid K	-	*G. lucidum*	[176]
**18.**	Ethyl ganoderate J	-	*G. lucidum*	[176]
**19.**	Ganoderic acid D	Cytotoxicity against HeLa cells (17.3 μM), 5*α*-reductase inhibition—NE	*G. lucidum*, *G. applanatum*, *G. tsugae*	[156,165, 177,178,179]
**20.**	Ganoderic acid F	Cytotoxicity against HeLa cells (19.5 μM)	*G. lucidum*	[177,179]
**21.**	Ganoderic acid E	Cytotoxicity against tumor cell lines [Hep G2 (1.44 × 10^−4^ μM), HepG2,2,15 (1.05 × 10^−4^ μM), κB—NE, CCM2 (31.25 μM), p388 (5.02 μΜ)]	*G. lucidum*, *G. tsugae*	[165,177, 180]
**22.**	Ganoderic acid Df	Human aldose reductase inhibitory activity (22.8 μM/mL)	*G. lucidum*	[162]
**23.**	Ganosporeric acid A	-	*G. lucidum*	[181]
**24.**	Ganohainanic acid A	Cytotoxicity—NE	*G. hainanense*	[62]
**25.**	Acetyl ganohainanic acid A	Cytotoxicity—NE	*G. hainanense*	[62]
**26.**	Ganohainanic acid B	Cytotoxicity—NE	*G. hainanense*	[62]
**27.**	Ganohainanic acid C	Cytotoxicity—NE	*G. hainanense*	[62]
**28.**	Ganohainanic acid D	Cytotoxicity—NE	*G. hainanense*	[62]
**29.**	Acetyl ganohainanic acid D	Cytotoxicity—NE	*G. hainanense*	[62]
**30.**	Methyl ganoderate D	-	*G. lucidum*	[182,183]
**31.**	Methyl ganoderate E	-	*G. lucidum*	[184]
**32.**	Methyl ganoderate F	Inhibitory effects on EBV-EA induction (289 mol ratio/32 pmol TPA)	*G. lucidum*	[184,185]
**33.**	12*β*-Acetoxy-3*β*,7*β*-dihydroxy-11,15,23-trioxolanost-8-en-26-oic acid butyl ester	Antimicrobial [*Staphylococcus* *aureus* ATCC 6538 (68.5 μM) and *Bacillus subtilis* ATCC6633 (123.8 μM)]	*G. lucidum*	[186]
**34.**	12*β*-acetoxy-3,7,11,15,23-pentaoxolanost-8-en-26-oic acid butyl ester	Antimicrobial—NE	*G. lucidum*	[186]
**35.**	n-Butyl ganoderate H	Selective cholinesterase inhibition	*G. lucidum*	[154]
**36.**	Butyl ganoderate A	Cytotoxicity against 3T3-L1 cells —NE	*G. lucidum*	[183]
**37.**	Butyl ganoderate B	Cytotoxicity against 3T3-L1 cells —NE	*G. lucidum*	[183]
**38.**	3*β*,7*β*,15*β*-Trihydroxy-11,23-dioxo-lanost- 8,16-dien-26-oic acid	Anti-AChE—NE	*G. tropicum*	[187]
**39.**	3*β*,7*β*,15*β*-Trihydroxy- 11,23-dioxo-lanost-8,16- dien-26-oic acid methyl ester	Anti-AChE (15.72%)	*G. tropicum*	[187]
**40.**	3*β*,15*β*-Dihydroxy- 7,11,23-trioxo-lanost- 8,16-dien-26-oic acid methyl ester	Anti-AChE—NE	*G. tropicum*	[187]
**41.**	Ganoderenic acid G	-	*G. applanatum*	[188]
**42.**	Ganoderenic acid F	-	*G. applanatum*	[188]
**43.**	Methyl ganoderate I	-	*G. applanatum*	[188]
**44.**	Ganodermacetal	Toxic activity against brine shrimp larvae	*G. amboinense*	[153]
**45.**	Methyl ganoderate A acetonide	Anti-AChE (18.35 μM), anti-BChE—NE	*G. lucidum*	[154]
**46.**	Ganoderic acid Z	Cytotoxicity	*G. lucidum*	[189]
**47.**	Ganoderic acid W	Cytotoxicity	*G. lucidum*	[189]
**48.**	Ganoderic acid V	Cytotoxicity	*G. lucidum*	[189]
**49.**	Ganoderic acid U	-	*G. lucidum*	[190]
**50.**	Ganoderic acid DM	5*α*-Reductase inhibition (10.6 μM), anti-androgen and anti-proliferative activities, osteoclastogenesis inhibitor, inhibits prostate cancer cell growth, inhibits breast cancer cell growth	*G. lucidum*, *G. sinense*	[107,157, 158,160, 191,192]
**51.**	7-Oxo-ganoderic acid Z_2_	-	*G. resinaceum*	[193]
**52.**	7-Oxo-ganoderic acid Z_3_	-	*G. resinaceum*	[193]
**53.**	Ganoderic acid GS-1	Anti-HIV protease (58 µM)	*G. sinense*	[192]
**54.**	Ganoderic acid GS-2	Anti-HIV protease (30 μM)	*G. sinense*	[192]
**55.**	Ganoderic acid GS-3	Anti-HIV protease—NE	*G. sinense*	[192]
**56.**	Ganoderic acid Ma	-	*G. lucidum*	[194]
**57.**	Ganoderic acid Mb	-	*G. lucidum*	[194]
**58.**	Ganoderic acid Mc	-	*G. lucidum*	[194]
**59.**	Ganoderic acid Md	-	*G. lucidum*	[194]
**60.**	Ganoderic acid Mg	-	*G. lucidum*	[190]
**61.**	Ganoderic acid Mh	-	*G. lucidum*	[190]
**62.**	Ganoderic acid Mi	-	*G. lucidum*	[190]
**63.**	Ganoderic acid Mj	-	*G. lucidum*	[190]
**64.**	3*α*,22*β*-Diacetoxy-7*α*-hydroxyl-5*α*-lanost-8,24*E*-dien-26-oic acid	Cytotoxicity against 95D (IC_50_ = 23 μM/mL), HeLa human tumor cell lines (IC_50_ = 14.7 μM/mL)	*G. lucidum* (mycelia)	[195]
**65.**	7-O-Ethyl ganoderic acid O	Cytotoxicity against 95D (46.7 μM), HeLa cells (59.1 μM)	*G. lucidum*	[196]
**66.**	Ganorbiformin B	-	*G. orbiforme*	[36]
**67.**	Ganorbiformin C	-	*G. orbiforme*	[36]
**68.**	Ganorbiformin D	Cytotoxicity (against NCIH187, MCF-7, and κB—NE), nonmalignant Vero cells, antimalarial, antitubercular—NE	*G. orbiforme*	[36]
**69.**	Ganorbiformin E	Cytotoxicity against NCIH187 (70 µM), MCF-7, κB—NE, nonmalignant Vero cells, antimalarial, antitubercular—NE	*G. orbiforme*	[36]
**70.**	Ganorbiformin F	Cytotoxicity against NCIH187 (44 µM), MCF-7—NE and κB (63 µM), nonmalignant Vero cells (36 µM), antimalarial, antitubercular—NE	*G. orbiforme*	[36]
**71.**	7*β*,23*ξ*-Dihydroxy-3,11,15-trioxolanosta-8,20*E*(22)-dien-26-oic acid	-	*G. applanatum*	[38]
**72.**	Methyl ganoderenate D	-	*G. applanatum*	[38]
**73.**	3*β*,7*β*,20,23*ξ*-Tetrahydroxy-11,15- dioxolanosta-8-en-26-oic acid	-	*G. applanatum*	[38]
**74.**	7*β*,20,23*ξ*-Trihydroxy-3,11,15- trioxolanosta-8-en-26-oic acid	-	*G. applanatum*	[38]
**75.**	Ganoderic acid L	-	*G. lucidum*	[197]
**76.**	Methyl ganoderate L	-	*G. lucidum*	[197]
**77.**	Ganolucidic acid γa	PXR-mediated CYP3A4 expression—NE	*G. sinense*	[198]
**78.**	Ganolucidate F	PXR-mediated CYP3A4 expression	*G. sinense*	[198]
**79.**	Ganolucidic acid D	Cytotoxicity on tumor growth cells—NE	*G. lucidum*	[197,199]
**80.**	Methyl ganolucidate D	-	*G. lucidum*	[197]
**81.**	Hainanic acid A	Cytotoxicity—NE	*G. hainanense*	[62]
**82.**	Hainanic acid B	Cytotoxicity—NE	*G. hainanense*	[62]
**83.**	Ganoderic acid γ	Cytotoxicity against tumor cell growth Meth-A (ED_50_ = 15.6 μg/mL), LLC—NE	*G. lucidum*	[199]
**84.**	Ganoderic acid δ	Cytotoxicity against tumor cell growth Meth-A and LLC—NE	*G. lucidum*	[199]
**85.**	Ganoderic acid ε	Cytotoxicity against tumor cell growth Meth-A (ED_50_ = 12.2 μg/mL), LLC—NE	*G. lucidum*	[199]
**86.**	Ganoderic acid ζ	Cytotoxicity against tumor cell growth Meth-A and LLC—NE	*G. lucidum*	[199]
**87.**	Ganoderic acid η	Cytotoxicity against tumor cell growth Meth-A and LLC—NE	*G. lucidum*	[199]
**88.**	Ganoderic acid θ	Cytotoxicity against tumor cell growth Meth-A (ED_50_ = 5.7 μg/mL), LLC (ED_50_ = 15.2 μg/mL)	*G. lucidum*	[199]
**89.**	Ganoderiol G	-	*G. lucidum*	[200]
**90.**	Ganoderiol H	-	*G. lucidum*	[200]
**91.**	Ganoderiol I	-	*G. lucidum*	[200]
**92.**	24*S*,25*R*-Dihydroxy-3,7-dioxo-8-en-5*α*-lanost-26-ol	Cytotoxicity—NE	*G. hainanense*	[62]
**93.**	Ganoderone A	Antiviral: influenza A—NE, HSV (0.3 μg/mL)	*G. pfeifferi*	[46]
**94.**	Ganoderone C	Antiviral—NE	*G. pfeifferi*	[46]
**95.**	3,7,24-Trioxo-5*α*-lanost- 8,25-dien-26-ol	Cytotoxicity against HL-60 (15.70 µM), SMMC-7721 (15.52 µM), A-549 (15.81 µM), MCF-7 (20.08 µM), SW480—NE	*G. hainanense*	[62]
**96.**	Hainanaldehyde A	Cytotoxicity—NE	*G. hainanense*	[62]
**97.**	21-Hydroxy-3,7-dioxo-5*α*-lanost-8,24*E*-dien-26-ol	Cytotoxicity—NE	*G. hainanense*	[62]
**98.**	3*β*,11*α*-Dihydroxy-7-oxo-5*α*-lanost-8,24*E*-dien-26-ol	Cytotoxicity—NE	*G. hainanense*	[62]
**99.**	Lucialdehyde D	-	*G. lucidum*, *G. pfeifferi*	[46,201, 202]
**100.**	Ganoderiol J	-	*G. sinense*	[198]
**101.**	16*α*,26-Dihydroxylanosta-8,24-dien-3-one	Cytotoxicity against K-562 cells (13.3 μg/mL)	*G. hainanense*	[75]
**102.**	Lucidadiol	Antiviral: influenza virus type A (ED_50_ = 0.22 mmol/L), HSV—NE	*G. lucidum*, *G. pfeifferi*	[203,204]
**103.**	Sinensoic acid	-	*G. sinense*	[205]
**104.**	Tsugaric acid C	Cytotoxicity—NE	*G. tsugae*	[206]
**105.**	Colossolactone A	Moderate cytotoxicity against L-929, K-562, HeLa cells—NE, anti-inflammatory properties	*G. colossum*	[207]
**106.**	Ganoderenicfy A	Promoting angiogenesis activities	*G. applanatum*	[208]
**107.**	Colossolactone I	Moderate cytotoxicity against HCT-116 colorectal cancer cells, Antimalarial: *Plasmodium falciparum*—NE	*G. colossum*	[92,209, 210]
**108.**	Colossolactone II	Low cytotoxicity against HCT-116 colorectal cancer cells	*G. colossum*	[92,209]
**109.**	Ganodermalactone E	Antimalarial: *Plasmodium falciparum*—NE	*G. colossum*	[210]
**110.**	Colossolactone B	Moderate cytotoxicity against L-929, K-562, and HeLa cells, antimicrobial—NE, antibacterial—NE	*G. colossum*	[207,210, 211]
**111.**	Methyl ganolucidate A	-	*G. lucidum*	[170,174]
**112.**	Methyl ganolucidate B	-	*G. lucidum*	[170,174]
**113.**	Methyl ganoderate A	-	*G. lucidum*	[182]
**114.**	Methyl ganoderate B	-	*G. lucidum*	[182]
**115.**	Methyl ganoderate C	-	*G. lucidum*	[182]
**116.**	Methyl ganoderate C_2_	-	*G. lucidum* (dried fruit bodies)	[212]
**117.**	Compound B_8_	-	*G. lucidum* (dried fruit bodies)	[212]
**118.**	3*β*-Oxo-formyl-7*β*,12*β*-dihydroxy-5*α*-lanost-11,15,23-trioxo-8-en(*E*)-26-oic acid	-	*G. lucidum* (fruit bodies)	[213]
**119.**	Ganoderic acid B_8_	Cytotoxicity against LLC—NE, T47-D—NE, S-180—NE, Meth-A—NE	*G. lucidum* *(*fruit bodies)	[214]
**120.**	Ganoderic acid C_1_	Inhibitory activity against HIV-PR (0.18 mM)	*G. lucidum* (fruit bodies)	[168,214]
**121.**	12*β*-Acetoxy-3,7,11,15,23-pentaoxo-5*α*-lanosta-8-en-26-oic acid ethyl ester	Cytotoxicity against human HeLa cervical cancer cell lines (63 μM)	*G. lucidum*	[215]
**122.**	3*β*,7*β*-Dihydroxy-12*β*-acetoxy-11,15,23-trioxo-5*α*-lanosta-8-en-26-oic acid methyl ester	-	*G. lucidum*	[32]
**123.**	3*β*-Hydroxy-7,11,12,15,23-pentaoxolanost-8-en-26-oic acid	Cytotoxic against p388 cell (9.85 μM), HeLa cell (17.10 μM), BEL-7402 cell (51.00 μM), SGC-7901 cells (42.00 μM)	*G. lucidum* (fruit bodies)	[216]
**124.**	Ganoderic acid H	Inhibitory activity against HIV-PR (0.20 mM)	*G. lucidum* (fruit bodies)	[177,217]
**125.**	Ganoderic acid K	Cytotoxicity against p388 cell (13. 8 μM), HeLa cell (8.23 μM); BEL-7402 cell (16.5 μM), SGC-7901cell (21.0 μM)	*G. lucidum* (fruit bodies)	[218]
**126.**	Ganoderic acid AM_1_	Cytotoxicity against p388 cell (13. 2 μM), HeLa cell (9.75 μM), BEL-7402 cell (20.9 μM), SGC-7901 cell (23.0 μM)	*G. lucidum* (fruit bodies)	[218]
**127.**	Ganoderic acid J	Cytotoxicity against p388 cell (15. 8 μM), HeLa cell (12.2 μM), BEL-7402 cell (25.2 μM), SGC-7901 cell (20.2 μM)	*G. lucidum* (fruit bodies)	[218]
**128.**	Ganoderic acid AP_2_	-	*G. applanatum* (fruit bodies)	[178]
**129.**	23*S*-Hydroxy-3,7,11,15-tetraoxolanost-8,24*E*-diene-26-oic acid	Cytotoxicity against p388 cell (15.7 μM), HeLa cell (9.72 μM), BEL-7402 cell (25.6 μM), SGC-7901 cell (23.1 μM)	*G. lucidum* (fruit bodies)	[218]
**130.**	7-Oxoganoderic acid Z	Inhibitory activities against the HMG-CoA reductase (22.3 μM), acyl CoA acyltransferase (5.5 μM)	*G. lucidum* (fruit bodies)	[219]
**131.**	Ganoderic acid LM_2_	Potent enhancement of ConA-induced mice splenocytes proliferation in vitro	*G. lucidum* (fruit bodies)	[220]
**132.**	Lucialdehyde B	Cytotoxic effect on tested tumor cells	*G. lucidum* (fruit bodies)	[214]
**133.**	Lucialdehyde C	Cytotoxicity against LLC, T-47D (10.7 µg/mL), Sarcoma 180 (4.7 µg/mL), Meth-A tumor cells (3.8 µg/mL)	*G. lucidum* (fruit bodies)	[214]
**134.**	Ganoderic acid *β*	HIV-I protease inhibitory activity (20 µM)	*G. lucidum* (spores)	[221]
**135.**	Ganolucidic acid E	-	*G. lucidum* (fruit bodies)	[200]
**136.**	Ganoderal B	-	*G. lucidum*	[222]
**137.**	11*α*-Hydroxy-3,7-dioxo-5*α*-lanosta-8,24(*E*)-dien-26-oic acid	Cytotoxicity against human HeLa cervical cancer cell lines (123 μM)	*G. lucidum*	[215]
**138.**	11*β*-Hydroxy-3,7-dioxo-5*α*-lanosta-8,24(*E*)-dien-26-oic acid	Cytotoxicity against human HeLa cervical cancer cell lines (51 μM)	*G. lucidum*	[215]
**139.**	Lucidal	-	*G. lucidum* (cultured fruit bodies)	[203]
**140.**	Lucialdehyde E	Cytotoxic activity against esophageal tumor EC109 cell line (18.7 mg/mL)	*G. lucidum* (spores)	[202]
**141.**	3*α*,22*β*-Diacetoxy-7*α*-hydroxyl-5*α*-lanost-8,24*E*-dien-26-oic acid	Cytotoxicity against HeLa cell lines (14.7 μM), 95D cell lines (23.01 μM)	*G. lucidum* (mycelial mat)	[195]
**142.**	Ganoderic acid O	-	*G. lucidum* (cultured mycelium)	[223]
**143.**	7-O-Methylganoderic acid O	-	*G. lucidum* (cultured mycelium)	[223]
**144.**	12*β*-Acetoxy-3*β*-hydroxy-7,11,15,23- tetraoxo-lanost-8,20*E*-diene-26-oic acid	Cytotoxicity against human cancer cell p388 (12.7 µM), HeLa cell (8.72 µM), BEL-7402 (24.2 µM), SGC-7901 (18.7 µM)	*G. lucidum* (fruit bodies)	[218]
**145.**	23-Dihydroganoderenic acid D	-	*G. applanatum* (fruit bodies)	[38]
**146.**	Ganoderenic acid A	-	*G. lucidum* (dried fruit bodies)	[177]
**147.**	Ganoderenic acid B	-	*G. lucidum* (dried fruit bodies)	[177]
**148.**	Ganoderenic acid C	-	*G. lucidum* (dried fruit bodies)	[177]
**149.**	Ganoderenic acid D	-	*G. lucidum* (dried fruit bodies)	[177]
**150.**	12*β*-Acetoxy-7*β*-hydroxy-3,11,15,23- tetraoxo-5*α*-lanosta-8,20-dien-26-oic acid	Cytotoxicity against human HeLa cervical cancer cell lines—NE	*G. lucidum*	[215]
**151.**	Methy ganoderenate H	-	*G. applanatum* (fruit bodies)	[188]
**152.**	Methyl ganoderenate I	-	*G. applanatum* (fruit bodies)	[188]
**153.**	12*β*-Acetoxy-3*β*,7*β*-dihydroxy-11,15,23-trioxo-5*α*-lanosta-8,20-dien-26-oic acid	-	*G. lucidum*	[32]
**154.**	Methyl ganoderate G	-	*G. lucidum*	[170]
**155.**	Compound C5	-	*G. lucidum* (fruit bodies)	[217]
**156.**	Compound C6	-	*G. lucidum* (fruit bodies)	[217]
**157.**	Ganoderic acid AP_3_	-	*G. applanatum* (fruit bodies)	[178]
**158.**	23-Dihydroganoderic acid I	-	*G. applanatum* (fruit bodies)	[38]
**159.**	23-Dihydroganoderic acid N	-	*G. applanatum* (fruit bodies)	[38]
**160.**	20-Hydroxylganoderic acid G	-	*G. lucidum* (fruit bodies)	[224]
**161.**	Lucidumol A	HIV-I protease inhibitory activity—NE	*G. lucidum* (spores)	[221]
**162.**	Ganoderiol C	-	*G. lucidum* (fruit bodies)	[200]
**163.**	Ganoderiol D	-	*G. lucidum* (fruit bodies)	[200]
**164.**	Ganoderitriol M	-	*G. lucidum* (fruit bodies)	[225]
**165.**	Tsugaric acid A	-	*G. tsugae*	[226]
**166.**	Tsugarioside A	Cytotoxicity against PLC/PRF/5 (ED_50_ = 6.5 μg/mL), T-24 (ED_50_ = 8.6 μg/mL), HT-3 (ED_50_ = 7.2 μg/mL), SiHa (ED_50_ = 9.5 μg/mL)	*G. tsugae* (fruit bodies)	[206]
**167.**	3-Oxo-5*α*-lanosta-8,24-dien-21-oic acid	Cytotoxicity—NE	*G. resinaceum* (fruit bodies)	[227]
**168.**	3*β*-Hydroxy-5α-lanosta-8,24-dien-21-oic acid	Cytotoxicity against T-24 (ED_50_ = 4.4 μg/mL), HT-3 (ED_50_ = 3.5 μg/mL), SiHa (ED_50_ = 5.5 μg/mL), CaSKi (ED_50_ = 6.2 μg/mL)	*G. tsugae* (fruit bodies)	[206]
**169.**	3*β*,7*β*-Dihydroxy-11,15,23-trioxolanost-8,16-dien-26-oic acid	-	*G. lucidum* (fruit bodies)	[203]
**170.**	3*β*,7*β*-Dihydroxy-11,15,23-trioxolanost-8,16-dien-26-oic acid methyl ester	-	*G. lucidum* (fruit bodies)	[203]
**171.**	12*β*-Acetoxy-3*β*,7*β*-dihydroxy-11,15,23-trioxolanost-8,16-dien-26-oic acid	-	*G. lucidum* (fruit bodies)	[228]
**172.**	Methyl ganoderate AP	-	*G. applanatum* (fruit bodies)	[188]
**173.**	Ganoderiol E	-	*G. lucidum* (fruit bodies)	[200]
**174.**	Epoxyganoderiol A	-	*G. lucidum*	[222]
**175.**	3*α*-Carboxyacetoxy-24-methylene-23-oxolanost-8-en-26-oic acid	Cytotoxicity—NE	*G. applanatum* (fruit bodies)	[229]
**176.**	3*α*-Carboxyacetoxy-24-methyl-23- oxolanost-8-en-26-oic acid	Cytotoxicity—NE	*G. applanatum* (fruit bodies)	[229]
**177.**	3-Epipachymic acid	-	*G. resinaceum* (fruit bodies)	[227]
**178.**	3*β*,15*α*-Diacetoxylanosta-8,24-dien-26-oic acid	-	*G. lucidum* (mycelia)	[230]
**179.**	Ganoderic acid V_1_	-	*G. lucidum*	[231]
**180.**	Tsugaric acid B	-	*G. tsugae*	[226]
**181.**	Methyl ganoderenate E	-	*G. lucidum* (fruit bodies)	[175]
**182.**	Lucidumol D	Selective anti-proliferative and cytotoxic effects	*G. lingzhi*	[232]
**183.**	Lucidumol C	Selective anti-proliferative and cytotoxic effects	*G. lingzhi*	[232]
**184.**	Leucocontextin A	-	*G. leucocontextum*	[233]
**185.**	Leucocontextin B	-	*G. leucocontextum*	[233]
**186.**	Leucocontextin C	-	*G. leucocontextum*	[233]
**187.**	Leucocontextin D	-	*G. leucocontextum*	[233]
**188.**	Leucocontextin E	-	*G. leucocontextum*	[233]
**189.**	Leucocontextin F	-	*G. leucocontextum*	[233]
**190.**	Leucocontextin G	-	*G. leucocontextum*	[233]
**191.**	Leucocontextin H	-	*G. leucocontextum*	[233]
**192.**	Leucocontextin I	-	*G. leucocontextum*	[233]
**193.**	Leucocontextin R	Cytotoxicity against K562 and MCF-7 cell lines (IC_50_ = 20–30 μM)	*G. leucocontextum*	[233]
**194.**	Ganoleuconin A	Cytotoxicity against K562 (17.8 μM), PC-3 cell lines—NE	*G. leucocontextum*	[34]
**195.**	Ganoleuconin B	Cytotoxicity against K562 (19.7 μM), PC-3 cell lines—NE	*G. leucocontextum*	[34]
**196.**	Ganoleuconin E	Cytotoxicity against K562 and PC-3 cell lines—NE	*G. leucocontextum*	[34]
**197.**	Ganoleuconin G	Cytotoxicity against K562 (11.4 μM), PC-3 cell lines (132.4 μM)	*G. leucocontextum*	[34]
**198.**	Ganoleuconin H	Cytotoxicity against K562 (115.4 μM), PC-3 cell lines (24.2 μM)	*G. leucocontextum*	[34]
**199.**	Ganoleuconin I	Cytotoxicity against K562 and PC-3 cell lines—NE	*G. leucocontextum*	[34]
**200.**	Ganoderenicfy B	Promoting angiogenesis activities	*G. applanatum*	[208]
**201.**	(*24E*)-15*α*,26-Dihydroxy-3-oxo-lanosta-8,24-diene	Antimycobacteria (50 μg/mL), cytotoxicity (5.9 μg/mL)	*G. casuarinicola*	[234]
**202.**	(*24E*)*-*7*α*,26-Dihydroxy-3-oxo-lanosta-8,24-diene.	Antimalarial activity (IC_50_ = 9.7 μg/mL)	*G. casuarinicola*	[234]
**203.**	(*24E*)*-*3-Oxo-7*α*,15*α*,26-trihy-droxylanosta-8,24-diene	Antimalarial activity (IC_50_ = 9.2 μg/mL)	*G. casuarinicola*	[234]
**204.**	(*24E*)*-*3*β*-Acetoxy-15α,26-dihydroxylanosta-8,24-diene	Antimycobacteria (25 μg/mL), cytotoxicity (6 μg/mL)	*G. casuarinicola*	[234]
**205.**	(*24E*)*-*3*β*-*A*cetoxy-7*α*,15*α*,26- trihydroxylanosta-8,24-diene	Antimycobacteria (25 μg/mL), cytotoxicity (9 μg/mL)	*G. casuarinicola*	[234]
**206.**	(*24E*)*-*3*β*,7*α*,15*α*,26-Tetra-hydroxylanosta-8,24-diene	-	*G. casuarinicola*	[234]
**207.**	7*β*,15*α*,20-Trihydroxy-3,11,23-trioxo-5α-lanosta-8-en-26-oic acid	-	*G. lucidum*	[235]
**208.**	Ganoderic acid XL_3_	-	*G. theaecolum*	[236]
**209.**	Ganoderic acid XL_4_	-	*G. theaecolum*	[236]
**210.**	Ganodecalone A	Cytotoxicity against K562 (17.22 µM)	*G. calidophilum*	[53]
**211.**	Ganoderic acid C_6_	-	*G. lucidum* (fruit bodies)	[65]
**212.**	Ganoderic acid D_1_	-	*G. lucidum* (fruit bodies)	[65]
**213.**	Ganoderic acid M	-	*G. lucidum* (fruit bodies)	[65]
**214.**	Ganoderic acid N	-	*G. lucidum* (fruit bodies)	[65]
**215.**	12-Hydroxylganoderic acid C_2_	-	*G. lucidum* (fruit bodies)	[65]
**216.**	3-Acetylganoderic acid H	-	*G. lucidum* (fruit bodies)	[65]
**217.**	12-Acetoxyganoderic acid D	-	*G. lucidum* (fruit bodies)	[65]
**218.**	12-Hydroxyganoderic acid D	-	*G. lucidum* (fruit bodies)	[65]
**219.**	12-Acetoxyganoderic acid F	-	*G. lucidum* (fruit bodies)	[65]
**220.**	12*β*-Hydroxy-3,7,11,15,23-pentaoxo-5*α*-lanosta-8-en-26-oic acid	-	*G. lucidum* (fruit bodies)	[65]
**221.**	12-Hydroxy-3,7,11,15,23-pentaoxo-lanost-8-en-26-oic acid	-	*G. lucidum* (fruit bodies)	[65]
**222.**	12,15-Bis(acetyloxy)-3-hydroxy-7,11,23-trioxo-lanost-8-en-26-oic acid	-	*G. lucidum* (fruit bodies)	[65]
**223.**	Methyl ganoderate J	-	*G. lucidum* (fruit bodies)	[65]
**224.**	Methyl-O-acetylganoderate C	-	*G. lucidum* (fruit bodies)	[65]
**225.**	Methyl ganoderate C1	-	*G. lucidum* (fruit bodies)	[65]
**226.**	Methyl ganoderate AM	-	*G. lucidum* (fruit bodies)	[65]
**227.**	Ganoderic aldehyde A	-	*G. lucidum* (fruit bodies)	[65]
**228.**	Ganoderenic acid K	-	*G. lucidum* (fruit bodies)	[65]
**229.**	Ganoderenic acid E	-	*G. lucidum* (fruit bodies)	[65]
**230.**	Elfvingic acid A	-	*G. lucidum* (fruit bodies)	[65]
**231.**	12*β*-Acetoxy-3*β*,7*β*-dihydroxy-11,15,23-trioxo-5*α*-lanosta-8,20-dien-26-oic acid	-	*G. lucidum* (fruit bodies)	[65]
**232.**	Methyl ganolucidate C	-	*G. lucidum* (fruit bodies)	[65]
**233.**	Ganolucidic acid C	-	*G. lucidum* (fruit bodies)	[65]
**234.**	Ganoderic acid C_2_	-	*G. lucidum* (fruit bodies /spore)	[65]
**235.**	Ganodrol A	Moderately inhibits FAAH (Inhibition rate in between 50–60%)	*G. lucidum*	[128]
**236.**	Ganodrol C	Moderately inhibits FAAH (Inhibition rate in between 50–60%)	*G. lucidum*	[128]
**237.**	Ganodrol D	Moderately inhibits FAAH (Inhibition rate in between 30–40%)	*G. lucidum*	[128]
**238.**	Ganoderic acid XL_5_	Cytotoxicity against human tumor cell lines—NE	*G. theaecolum*	[236]
**239.**	Methyl gibbosate M	Anti-adipogenesis activity—NE	*G. applanatum*	[51]
**240.**	Methyl ganoapplate E	Anti-adipogenesis activity—NE	*G. applanatum*	[51]
**241.**	Applandiketone A	-	*G. applanatum*	[237]
**242.**	Applandiketone B	Significant inhibitory effect against NO production in LPS-induced RAW264.7 cells (IC_50_ = 20.65 µM)	*G. applanatum*	[237]
**243.**	15*α*-Acetoxy-3*α*-hydroxylanota- 8,24-dien-26-oic	-	*G. capense*	[238]
**244.**	Ganoderterpene A	Strongly suppressed NO generation in BV-2 microglial cells treated with lipopolysaccharide (LPS) (IC_50_ = 7.15 µM), significantly suppressed the activation of MAPK and TLR-4/NF-κB signaling pathways, effectively improved the LPS-induced mitochondrial membrane potential and apoptosis	*G. lucidum*	[239]
**245.**	Ganodeweberiol G	Significant *α*-glucosidase inhibitory activity (IC_50_ = 165.9 µM)	*G. weberianum*	[77]

Remake: NE = No Effect.

##### 8,9-dihydro-triterpenoids

**Figure 5:**
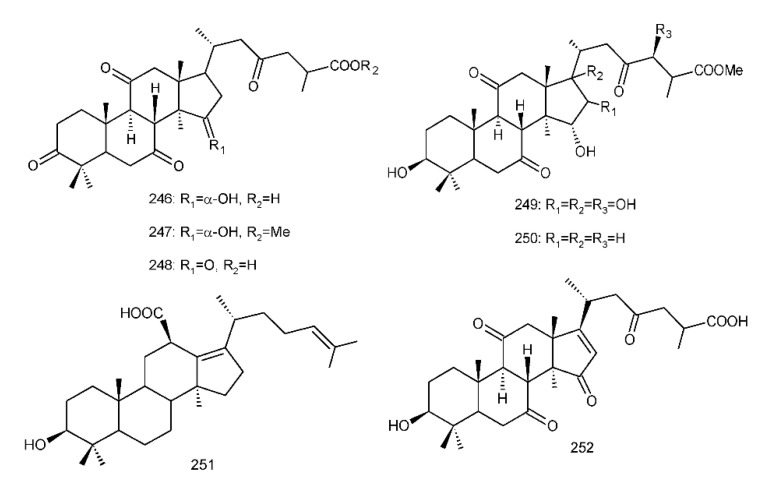
Chemical structures of several 8,9-dihydro-triterpenoids (**246**–**252**).

From a lipophilic extract of the fruiting body of *G. lucidum*, two dihydroganoderic acids, 8*β*,9*α*-dihydroganoderic acid J (**246**) and methyl 8*β*,9*α*-dihydroganoderate J (**247**)**,** (Figure 5) were first isolated [224]. Ma et al. [224] used Nuclear Overhauser Effect Spectroscopy (NOESY) experiments to confirm the absolute configuration of H-8 and H-9 as *β* and *α*. 8*β*,9*α*-Dihydroganoderic acid C (**248**) (Figure 5) was also isolated from this fungus [176]. Then, Peng et al. [193] studied the chemical constituents of *G. resinaceum*, and ganoderesins A and B (**249**, **250**) (Figure 5) were identified to be 8*β*,9*α*-dihydroganoderate derivatives. Meanwhile, in an in vitro model, compound **250** showed inhibitory effects against the increase in alanine aminotransferase (ALT) and aspartate aminotransferase (AST) levels in HepG2 cells induced using H_2_O_2_ compared to a control group in the range of its maximum non-toxic concentration. Fornicatin C (**251**) (Figure 5) from *G. fornicatum* also possessed an 8*β*,9*α*-dihydrolanostane skeleton. However, 18-COOH was shifted to C-12 and a double bond was present between C-13 and C-17 [61]. Liu et al. [74] isolated ganoderesin C (**252**) (Figure 5) from *G. theaecolum* with the 8*β*,9*α*-dihydrolanostane skeleton and exhibited hepatoprotective activity at a concentration of 10 µM.

##### 8,9-epoxy-triterpenoids

**Figure 6:**
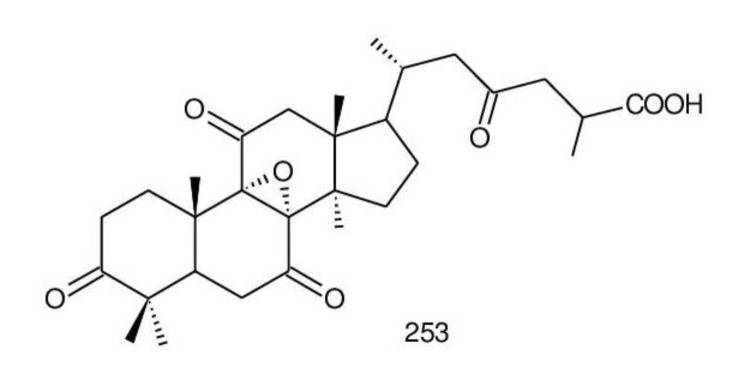
Chemical structure of 8*α*,9*α*-epoxy-3,7,11,15,23-pentaoxo-5*α*-lanosta-26-oic acid (**253**).

Lanostanol was formed through a MVA pathway as an 8,9-ene skeleton; nevertheless, the double bond was transformed to an epoxy group due to the presence of oxygenase’s [135]. 8*α*,9*α*-Epoxy-3,7,11,15,23-pentaoxo-5*α*-lanosta-26-oic acid (**253**) (Figure 6) was isolated from the chloroform extract of the fruiting bodies of *G. lucidum* and exhibited good antifungal activity against *Candida albicans* in disc diffusion assay (100 μg/disc) [240].

##### 7(8),9(11)-diene-triterpenoids

**Figure 7:**
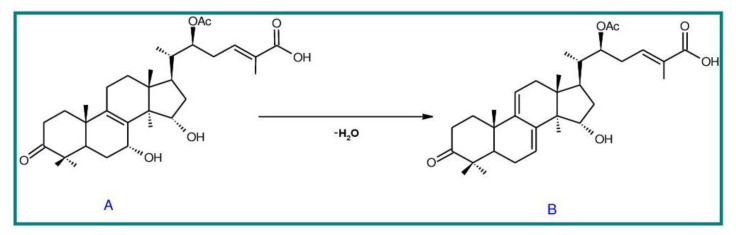
7(8),9(11)-diene-triterpenoids: compound (**A**) was transformed to compound (**B**).

Δ^7(8),9(11)^-triterpenoids are also a series of lanostane-type triterpenoids which were frequently isolated from *Ganoderma* (Table 2). Isaka et al. [36] investigated the mycelia extract from cell culture of *G. orbiforme*, stain BCC 22324 (received from Thailand) and a range of lanostane triterpenoids were isolated. When they used the ^1^H NMR technique (CDCl_3_ used as a solvent) to confirm the structures of compound A and its 7-*O*-acetate derivative, each compound was converted to the same 7,9(11)-diene skeleton B (Figure 7). A similar elimination reaction of 7*α*-OMe ganoderic acid derivatives under acidic conditions was previously reported [175,190,194].

Ganoderic acid Mf (**263**), ganoderic acid S (**273**) and ganoderic acid Mk (**312**) showed moderate cytotoxicity against 95D and HeLa human tumor cells [241,242], and their stability was studied with two pairs of double bonds at positions C-7(8) and 9(11) being observed [195]. 

**Figure 8:**
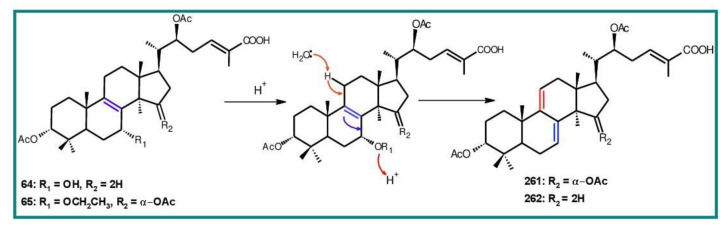
7(8),9(11)-diene-triterpenoids: compounds **64**, **65** decomposed to compounds **261**, **262** in the acid condition.

The stability of bioactive compounds was a key problem regarding its application and analysis. Stability-related research works have rarely been reported for ganoderic acids and the stability of triterpenoids in various conditions was systematically described in this research [195,243]. Ganoderic acid Md (**59**) that had a methoxy group at C-7 was documented to be converted into ganoderic acid R (**262**) using treatment of H_2_SO_4_ acid [194]. Also, Li et al. [195] reported ganoderic acid Mc (**58**) contains an acetyl group at the position of C-7 and it was readily converted to ganoderic acid Mk (**312**) in protic conditions. Also, ganoderic acid compounds (**263**, **273**, **312**) possess two pairs of double bonds at the positions of C-7(8) and 9(11) and even in the HCl–MeOH solution, and these compounds were shown rather stable. Wang et al. [243] showed that unsuitable pH (H^+^/OH^−^) and a high operating temperature are the key factors that affect the structure and properties of 7-O-ethyl ganoderic acid O (**65**), and Li et al. [195] concluded that the unstable property of 7-O-ethyl ganoderic acid O (**65**) occurs due to the ether bond at the C-7 position. However, 3*α*,22*β*-diacetoxy-7*α*-hydroxyl-5*α*-lanost-8,24*E*-dien-26-oic acid (**64**) degraded to ganoderic acid R (**262**) in the protic environment because of the hydroxyl group at the C-7 position [195] and showed a similar degradation of 7-O-ethyl ganoderic acid O (**65**) to ganoderic acid T (**261**) [243] (Figure 8).

Highly oxygenated triterpenoids have been identified from *Ganoderma*. Most interestingly, some of the species of *Ganoderma* can produce pairs of oxygenated triterpenoids that are C-3 *α*- or *β*-relative configuration triterpenoids, and C-3 or C-15 positional isomers such as compounds **256**/**257** and **264**/**265** [244]. Although the biogenesis of triterpenoids is obvious, it is interesting to know the biosynthetic pathway along which paired C-3 *α*/*β* stereoisomers and C-3/C-15 positional isomers are produced [244]. Further experiments also verified the transformation of 3*β* into 3*α* of triterpenoid when adding the labelled 3*β* triterpenoid into the liquid culture and 3*α* was derived from 3*β* series through an oxidation-reduction pathway. Multiple pairs of 3*α* stereoisomers are produced in cultured mycelia during the production period of the 3*β* triterpenoids. The corresponding 3-keto metabolite can also be identified in the mycelia [244].

Except for the OH-3*α*/*β* stereoisomers, ganoderic acid S_Z_ (**275**) isolated from a lipophilic extract of the fruiting body of *G. lucidum* is a geometric Z-isomer of tyromycic acid from *Tyromyces fissilis* [245,246]. This was confirmed by the NOESY cross peak of H-24/H_3_-27. However, their chemical shifts of C-24, C-25, C-26 and C-27 in the CDCl_3_ were the same. Li et al. [246] named compound **275** “ganoderic acid S”; nevertheless, the structure of ganoderic acid S should be compound **273**, which was first isolated from *G. lucidum* by Hirotani et al. [247]. 

**Figure 9:**
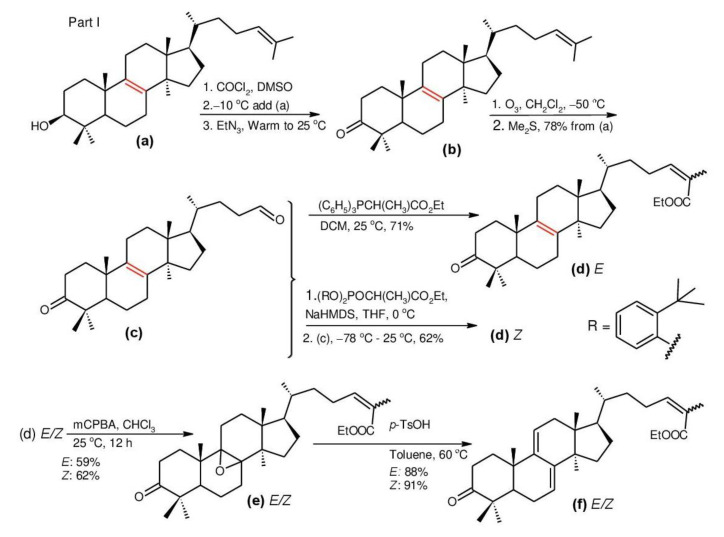

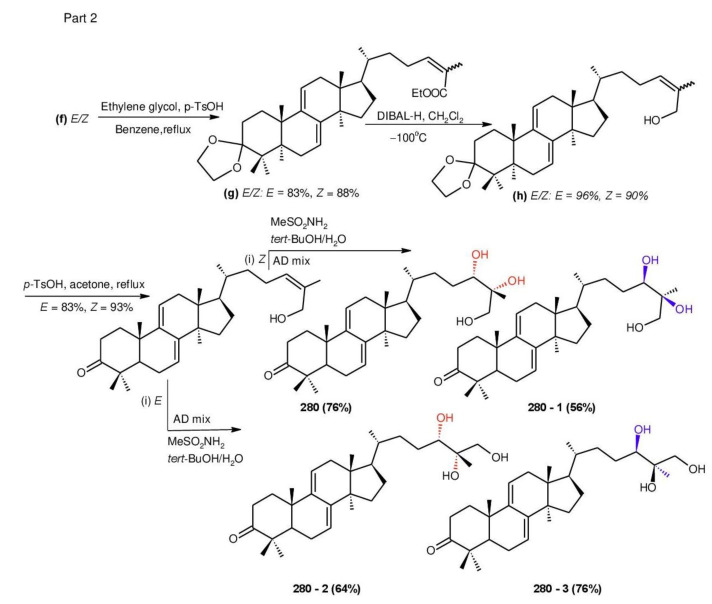
7(8),9(11)-diene-triterpenoids: semi-synthesis of ganodermantriol (**291**) and its stereoisomeric triols.

Ganodermanontriol (**291**) was a highly oxygenated lanostane-type triterpenoid and first isolated from *G. lucidum* [248]. Previous studies observed that ganodermanontriol (**291**) showed various biological activities, such as anti-HIV-1, anti-HIV protease and anti-complement [248]. Thus, Kennedy et al. [249] first obtained **291** and its stereoisomeric triols using semi-synthesis from lanosterol (a) over nine steps via the construction of the dienone core and elaboration (part 1) of the 7,9(11)-diene core to triols (part 2). The key steps leading to this family of isomers involved the reconstruction of the trisubstituted alkene using stereoselective and chemoselective phosphonate reactions and the formation of the unusual Δ^7,9(11)^-diene core using the mild acidic opening of a lanosterone-derived epoxide (Figure 9). Ganodermanontriol exhibited significant results on the inhibition and proliferation of breast cancer cells with inhibitory activity (IC_50_ = 5.8 μM at 72 h) on the proliferation of MCF-7 cancer cells and IC_50_ value of 9.7 μM for the MDA-MB-231 cell line [249]. 

Ganodercochlearin C (**293**)**,** ganodecochlearins A and B (**294** and **295**)**,** and 3*β*,22*S*-dihydroxylanosta-7,9(11)**,**24-triene (**297**) were isolated from the fruiting bodies of *G. cochlear* [57]. Compound **296** was an acetyl derivative of **295**. Among them, ganodecochlearins A and B (**294** and **295**) possessed a five-membered ether ring in the side chain and were first identified from *Ganoderma* [250]. However, their analogues, inonotsuoxides A and B with 22,25-epoxylanost-8-ene-3*β*,24*S*-diol skeleton, were isolated from the sclerotia of *Inonotus obliquus* [250]. 

A group of polyoxygenated lanostanoid triterpenoids, applanoxidic acids A (**298**)**,** B (**299**)**,** C (**302**)**,** D (**303**)**,** E (**300**), F (**301**), G (**304**) and H (**305**) were isolated from two Indonesian tropical macrofungi, *G. applanatum* and *G. annulare*. The structural characteristics of these compounds were the presence of 7,8-epoxy group, hydroxyl or carbonyl groups at C-12, as well as a hydroxyl group or double bond at C-20. The evaluation of their biological activities showed that all of them had weak anti-tumor activities [251,252] and applanoxidic acids A (**298**)**,** C (**302**), and F (**301**) exhibited antifungal activities against the fungi *Microsporum cannis* and *Trichophyton mentagrophytes* [48]. 

Enterovirus 71 (EV71) is a major causative agent for hand, foot, and mouth disease (HFMD), and a fatal neurological and systemic complicating agent in children [253]. However, this is currently not a clinically approved antiviral drug for the prevention and treatment of viral infection [253]. Ganoderic acid Y (**254**) and 5*α*-lanosta-7,9(11)**,**24-triene-15*α*-26-dihydroxy-3-one (**286**) were evaluated for their inhibitory effect against EV71, and both showed significant anti-EV71 activities without cytotoxicity in human rhabdomyosarcoma (RD) cells [253]. The mechanisms by which the two compounds affect EV71 infection were further elucidated by three action modes using Ribavirin, a common antiviral drug (positive control). The results suggested that compounds **254** and **286** can interact with the viral particle to block the adsorption of the virus to cells and this interaction was predicated using computer molecular docking [253]. The authors also demonstrated that compounds **254** and **286** significantly inhibit the replication of the viral RNA of Enterovirus 71 and inhibit EV71 replication via the blocking of EV71 uncoating [253]. 

Ganodermic acid S (GAS, **257**) is an interesting lanostane-type triterpenoid. GAS in incubated gel-filtered human platelets showed that it was more powerful in inhibiting U46619-activated platelet aggregation than aggregations activated using collagen or ADP-fibrinogen [254]. Further, GAS intensely hindered U46619-induced diacylglycerol formation, granule secretion, Ca^2+^ mobilization and arachidonic acid release [254]. GAS inhibited the collagen response predominantly for the TXA_2_-dependent signaling, and the tyrosine kinase-dependent pathway in collagen response plays a major role in aggregation [254,255]. However, their further research indicated that GA inhibits platelet response to TXA_2_ on the receptor-Gq-phospholipase C*β*1 pathway, but not on the receptor-G_1_ pathway [254]. Because of inhibitory effects on platelet responses to various aggregating agonists of GAS, it is also found that GAS participated in potentiating the response of human gel-filtered platelets to prostaglandin E_1_ [256]. 

Liu et al. [257] evaluated the effects of ganoderol B (**311**)**,** ganoderiol F (**284**) and ganodermanontriol (**291**) on the androgen receptor binding and the growth of LNCaP cells. The results showed that less than two hydroxyl groups in the 17*β*-side chain are needed for binding to an androgen receptor. In the case of the ganoderma alcohols with the same number of hydroxyl groups in the 17*β*-side chain, the one which has the C-3 carbonyl group showed better binding activity to androgen receptor than that which has the C-3 hydroxyl group. Among these compounds, ganodermanontriol (**289**) also inhibited invasive behaviour (cell adhesion, cell migration and cell invasion) through the suppression of secretion of urokinase-plasminogen activator (uPA) and inhibited expression of the uPA receptor, suggesting that this compound can be a natural agent for treating invasive breast cancers [258]. Ha et al. [259] confirmed that compound **291** exhibited in vitro and in vivo hepatoprotective activity as determined by the lowered levels of hepatic enzymes and malondialdehydes and the elevated glutathione levels.

**Figure 10:**
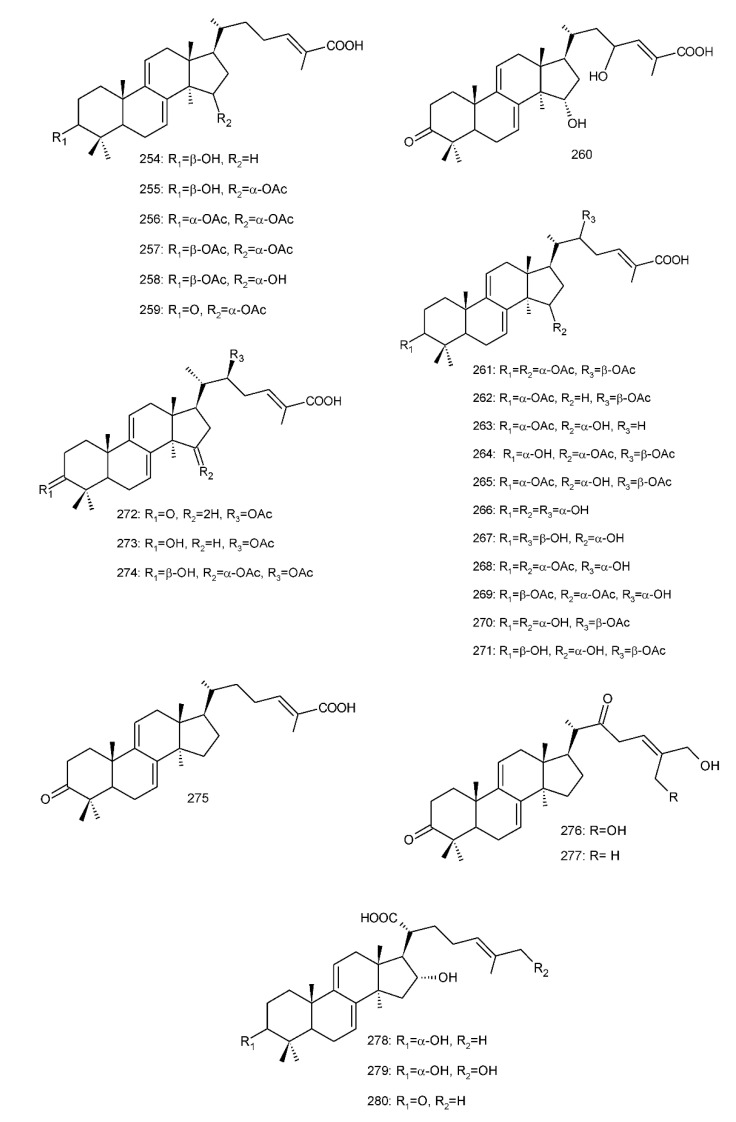

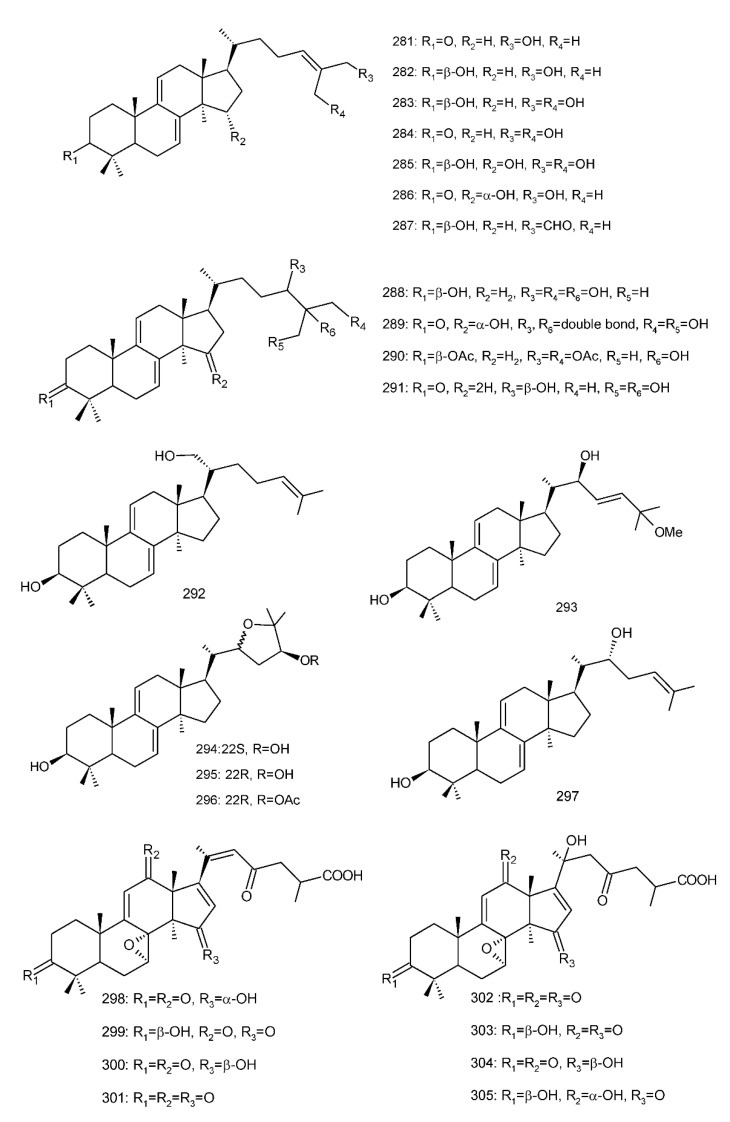

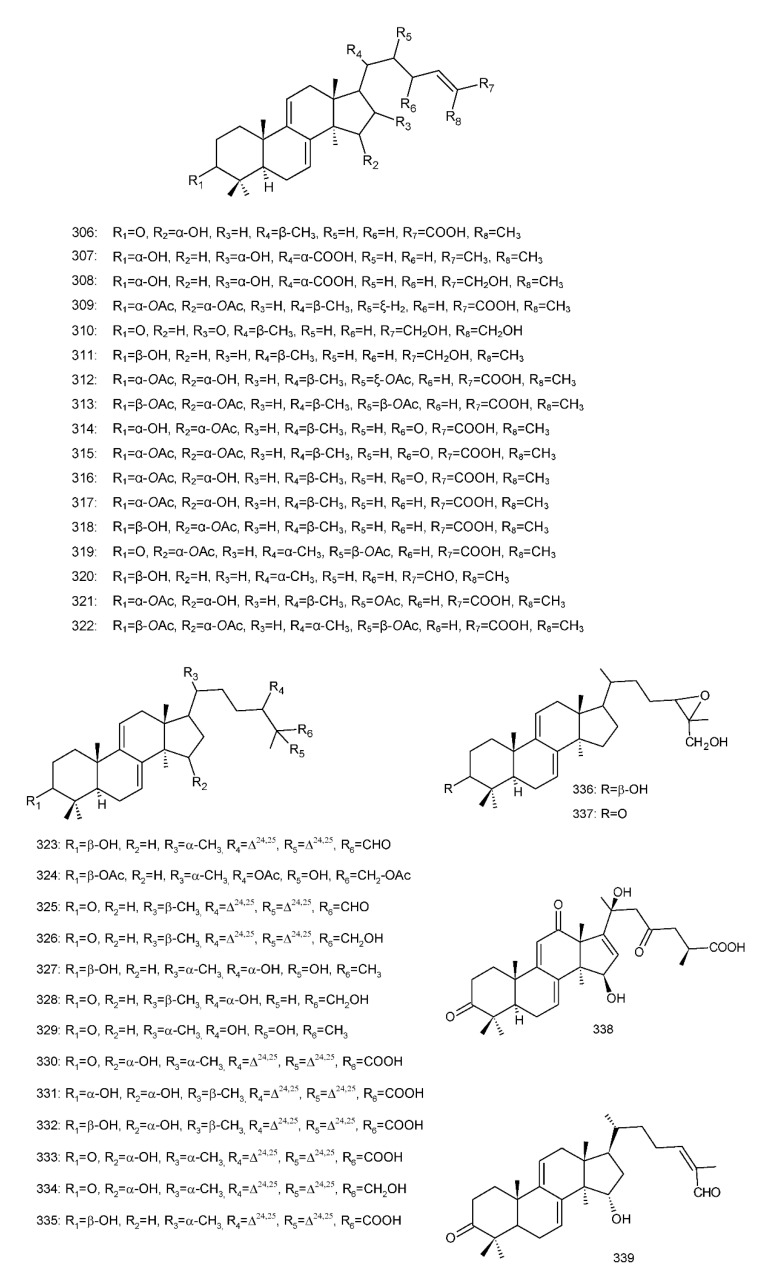

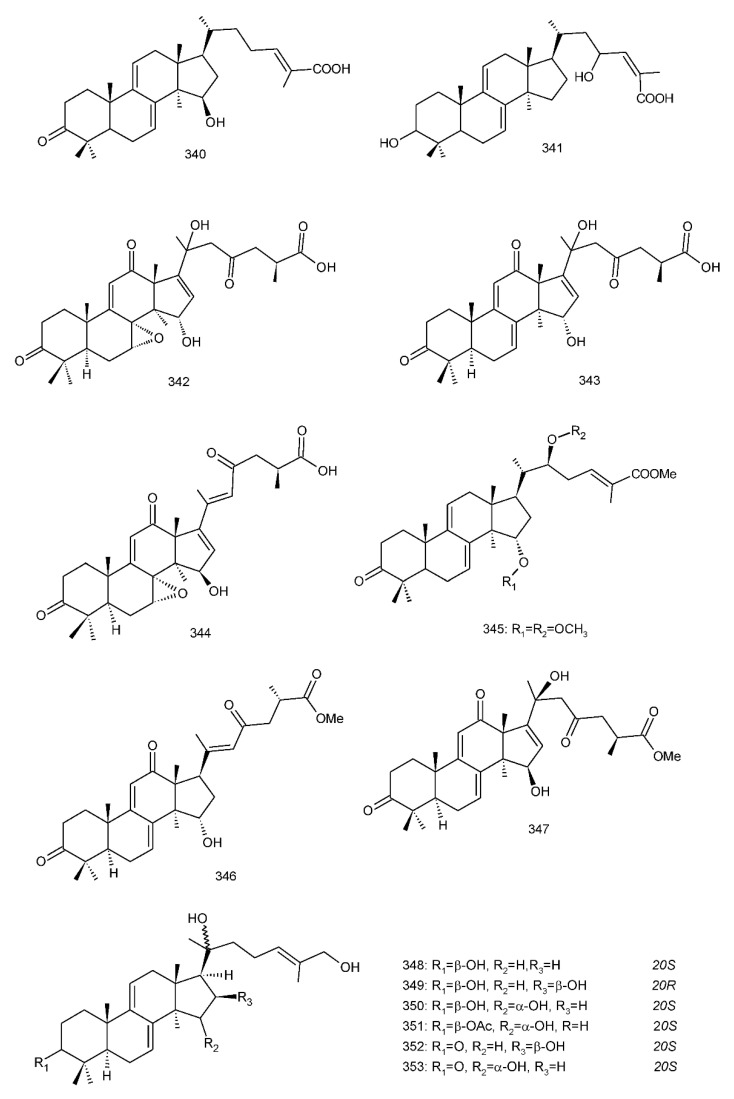
Chemical structures of several Δ7(8),9(11)-triterpenoids (**254**–**353**).

Chemical structures of different 7(8),9(11)-diene-triterpenoids are shown in Figure 10.

**Table 2:** Δ7(8),9(11)-triterpenoids and bioactivities from *Ganoderma*.

No.	Trivial Names	Bioactivities (IC_50_/MIC or ED_50_)	Sources *Ganoderma* Species	References
**254.**	Ganoderic acid Y	Mitogenesis, inhibition (0.180 mM), cholesterol production inhibition (1.40 µM), HMG-CoA reductase inhibition (8.60 µM), AChE inhibition (21.1 µM)	*G. lucidum*	[100,253]
**255.**	Ganoderic acid X	DNA Topoisomerase I/II inhibition	*G. lucidum*	[100,260]
**256.**	Ganodermic acid R	-	*G. lucidum*	[261,262]
**257.**	Ganodermic acid S	Induces aggregation of human platelet, inhibition of human platelet function, inhibits thromboxane A2-dependent pathway in human platelets response to collagen, differential effect on the thromboxane A2-signaling pathways in human platelets, potentiation on prostaglandin E1-induced cyclic AMP elevation in human platelets	*G. lucidum*	[262,263]
**258.**	Ganodermic acid T-O	-	*G. lucidum*	[264]
**259.**	Ganodermic acid T-Q	-	*G. lucidum*	[264]
**260.**	Ganoderic acid Jc	Cytotoxicity against HL-60 cells (8.30 μM)	*G. sinense*	[198]
**261.**	Ganoderic acid T	Anti-tumor (induce P53)	*G. lucidum*	[247,265]
**262.**	Ganoderic acid R	Strongly anti-hepatotoxic, multidrug resistance tumor cell line (κB-A1/Dox) and a sensitive tumor cell line (κB-A1)	*G. lucidum*	[247,266]
**263.**	Ganoderic acid Mf	-	*G. lucidum*	[194]
**264.**	Ganoderic acid P	-	*G. lucidum*	[223]
**265.**	Ganoderic acid Q	-	*G. lucidum*	[223]
**266.**	3*α*,15*α*,22*α*-Trihydroxylanosta-7,9(11),24-trien-26-oic acid	-	*G. lucidum*	[230]
**267.**	3*β*,15*α*,22*β*-Trihydroxylanosta-7,9(11),24-trien- 26-oic acid	-	*G. lucidum*	[230]
**268.**	3*β*,15*α*-Diacetoxy-22*α*-hydroxylanosta-7,9(11),24-trien-26-oic acid	-	*G. lucidum*	[230]
**269.**	3*α*,15*α*-Diacetoxy-22*α*-hydroxylanosta-7,9(11),24-trien-26-oic acid	-	*G. lucidum*	[230]
**270.**	22*β*-Acetoxy-3*α*,15*α*-dihydroxylanosta-7,9(11),24-trien-26-oic acid	-	*G. lucidum*	[230]
**271.**	22*β*-Acetoxy-3*β*,15*α*-dihydroxylanosta-7,9(11),24-trien-26-oic acid	-	*G. lucidum*	[230]
**272.**	Ganorbiformin G	Cytotoxicity against NCI-H187 (65.0 μM), MCF-7—NE, κB (65.0 μM), Vero (35.0 μM), antimalarial—NE, anti-TB—NE	*G. orbiforme*	[36]
**273.**	Ganoderic acid S	Cytotoxicity against NCI-H187 (39 μM), MCF-7—NE, κB (53.0 μM), Vero—NE, antimalarial—NE, anti-TB—NE, Strongly anti-hepatotoxic	*G. lucidum*, *G. orbiforme*	[36,247]
**274.**	Ganodermic acid P_2_	-	*G. lucidum*	[261]
**275.**	Ganoderic acid S_Z_	-	*G. lucidum*	[246]
**276.**	26,27-Dihydroxy-5*α*-lanosta-7,9(11),24-triene-3,22-dione	Induced NAD(P)H:quinone oxidoreductase (QR) in cultured hepalcic7 murine hepatoma cells (20.0 μg/mL)	*G. lucidum*	[267]
**277.**	26-Hydroxy-5*α*-lanosta-7,9(11),24-triene-3,22-dione	Induced NAD(P)H:quinone oxidoreductase (QR) in cultured hepalcic7 murine hepatoma cells (3 μg/mL)	*G. lucidum*	[267]
**278.**	3*α*,16*α*-Dihydroxylanosta-7,9(11),24-trien-21-oic acid	Cytotoxicity—NE	*G. applanatum*	[229]
**279.**	3*α*,16*α*,26-Trihydroxylanosta-7,9(11),24-trien-21-oic acid	Cytotoxicity—NE	*G. applanatum*	[229]
**280.**	16*α*-Hydroxy-3-oxolanosta-7,9(11),24-trien-21-oic acid	Cytotoxicity against P388 murine leukemia cells (111 μg/mL)	*G. applanatum*	[229]
**281.**	Ganodermenonol	Cytotoxicity against LLC—NE, T-47D (4.8 μg/mL), S-180 (10.0 μg/mL), Meth-A (2.8 μg/mL)	*G. lucidum*	[214,268]
**282.**	Ganodermadiol	Cytotoxicity against LLC—NE, T- 47D—NE, S-180—NE, Meth-A—10.3 μg/mL, protects Vero cells against HSV type 1 infection (ED_50_ = 0.068 mmol/L), protects MDCK cells against influenza virus type A infection (ED_50_ > 0.22 mmol/L)	*G. lucidum*, *G. pfeifferi*	[204,268]
**283.**	Ganodermatriol	Inhibition of 5*α*-reductase activity (%) at 667 μM (39%)	*G. lucidum*	[156,268]
**284.**	Ganoderiol F	Inhibition on cell growth in thepresence of testosterone or DHT, potential CDK4/CDK6 inhibitor for breast cancer therapy; anticomplementary activity (4.8 μM), inhibition of 5*α*-reductase—NE	*G. lucidum*, *G. leucocontextum*	[156,257, 269,270]
**285.**	Ganodermatetraol	Induction ability of hPXR-mediated CYP3A4 expression	*G. sinense*	[198]
**286.**	5*α*-Lanosta-7,9(11),24-triene- 15*α*-26-dihydroxy-3-one	Induces apoptosis in human promyelocytic leukemia HL-60 cells, inhibition of 5*α*-reductase activity (41.9 μM), antiviral (enterovirus 71)	*G. concinna*, *G. lucidum*	[156,253, 271]
**287.**	5*α*-Lanosta-7,9(11),24-triene- 3*β*-hydroxy-26-al	Induces apoptosis in human promyelocytic leukemia HL-60 cells	*G. concinna*	[271]
**288.**	Ganoderiol A	Suppresses migration and adhesion of MDA-MB-231 cells and minimal impact on cell invasion in MDA-MB-231 cells, 5*α*-reductase inhibitory activity—NE	*G. lucidum*	[156,272, 273]
**289.**	Ganoderiol B	Moderately active inhibitor against HIV-1 PR (0.17 mM), inhibition of 5*α*-reductase—NE	*G. lucidum*	[156,168, 272]
**290.**	3*β*,24,26-Triacetoxy-5*α*-lanosta-7,9(11)-dien-25-ol	-	*G. sinense*	[274]
**291.**	Ganodermanontriol	Anti-HIV-1 agent—(7.8 mg/mL), anti HIV protease; anticomplement activities, inhibition of 5*α*-reductase—NE	*G. lucidum*	[156,168, 248]
**292.**	Lanosta-7,9(11),24-trien-3*β*,21-diol	-	*G. australe*	[275]
**293.**	Ganodercochlearin C	-	*G. cochlear*	[57]
**294.**	Ganodercochlearin A	-	*G. cochlear*	[57]
**295.**	Ganodercochlearin B	-	*G. cochlear*	[57]
**296.**	Ganodecochlearin B diacetate	-	*G. cochlear*	[57]
**297.**	3*β*,22*S*-Dihydroxylanosta-7,9(11),24-triene	-	*G. cochlear*	[57]
**298.**	Applanoxidic acid A	Inhibitory effect on EBV-EA activation, antifungal activity against the growth of *Microsporum cannis* (1000 μg/mL), *Trichophyton mentagrophytes* (500 μg/mL), cytotoxicity against HL-60 cell line (132.0 μM)	*G. applanatum*, *G. australe*, *G. annulare*	[48,251, 252,276]
**299.**	Applanoxidic acid B	Remarkable inhibitory effect on EBV-EA activation	*G. applanatum*	[251,252]
**300.**	Applanoxidic acid E	Inhibitory effect on EBV-EA activation	*G. applanatum*	[252]
**301.**	Applanoxidic acid F	Inhibitory effect on EBV-EA activation, antifungal activity against the growth of *Microsporum cannis* (1000 μg/mL), *Trichophyton mentagrophytes* (1000 μg/mL), cytotoxicity against HL-60 cells (315.0 μM)	*G. applanatum*, *G. annular*, *G. australe*	[48,252, 276]
**302.**	Applanoxidic acid C	Inhibitory effect on EBV-EA activation, antifungal activity against the growth of *Microsporum cannis* (1000 μg/mL), *Trichophyton mentagrophytes* (1000 μg/mL), cytotoxicity against HL-60 cells (334.0 μM)	*G. applanatum*, *G. annulare*, *G. australe*	[48,251,252,276]
**303.**	Applanoxidic acid D	Inhibitory effect on EBV-EA activation	*G. applanatum*	[251,252]
**304.**	Applanoxidic acid G	Inhibitory effect on EBV-EA activation, antifungal—NE, antiviral—NE, cytotoxicity inhibits the viability and growth of the HL-60 cells (404.0 μM)	*G*.*applanatum*, *G. annulare*, *G. pfeifferi*, *G. australe*	[48,204, 252,276]
**305.**	Applanoxidic acid H	Inhibitory effect on EBV-EA activation	*G. applanatum*	[252]
**306.**	Ganoderic acid Jb	Inhibitory activities against the HMG-CoA reductase and acyl CoA acyltransferase	*G. lucidum* (fruit bodies)	[65,219]
**307.**	3*α*,16*α*-Dihydroxylanosta-7,9(11),24-trien-21-oic acid	Cytotoxicity against the P388 murine leukemia cell line—NE	*G. applanatum* (fruit bodies)	[137]
**308.**	3*α*,16*α*,26-Trihydroxylanosta-7,9(11),24-trien-21-oic acid	Cytotoxicity against the P388 murine leukemia cell line—NE	*G. applanatum* (fruit bodies)	[137]
**309.**	Ganoderic acid Me	-	*G. lucidum* (cultured mycelial mat)	[194]
**310.**	26,27-Dihydroxylanosta-7,9(11),24-trien-3,16-dione	-	*G. carnosum* (fruit bodies)	[55]
**311.**	Ganoderol B	-	*G* *. lucidum*	[277]
**312.**	Ganoderic acid Mk	-	*G. lucidum* (mycelial mat)	[190]
**313.**	Lanosta-7,9(11),24-trien-3*β*,15*α*, 22*β*-triacetoxy-26-oic acid	-	*G* *. lucidum*	[278]
**314.**	Lanosta-7,9(11),24-trien-15*α*-acetoxy-3*α*-hydroxy-23-oxo-26-oic acid	-	*G* *. lucidum*	[278]
**315.**	Lanosta-7,9(11),24-trien-3*α*,l5*α*-diacetoxy-23-oxo-26-oic acid	-	*G* *. lucidum*	[278]
**316.**	Lanosta-7,9(11),24-trien-3*α*,15*α*-hydroxy-23-oxo-26-oic acid	-	*G* *. lucidum*	[32]
**317.**	Lanosta-7,9(11),24-trien-3*α*-acetoxy-15*α*,22*β*-dihydroxy-26-oic acid	-	*G* *. lucidum*	[278]
**318.**	Ganodermic acid T-N	-	*G. lucidum* (mycelia)	[264]
**319.**	Compound 10	-	*G* *. orbiforme*	[36]
**320.**	5*α*-Lanosta-7,9(11),24-triene- 3*β*-hydroxy-26-al	Concentration of 30 μM induced apoptosis in 15% of the human promyelocytic leukemia HL-60 cell (after treatment for 24 h)	*G* *. concinna*	[271]
**321.**	Ganodermic acid P_1_	-	*G. lucidum* (mycelia)	[261]
**322.**	Lanosta-7,9(11),24-trien-3*β*,15*α*, 22-triacetoxy-26-oic acid	Concentration of 10 µg/mL showed toxicity towards the brine shrimp larvae (after treatment for 24 h)	*G. amboinense* (fruit bodies)	[153]
**323.**	Lucialdehyde A	Cytotoxicity against Meth-A (10.4 µg/mL)	*G. lucidum* (fruit bodies)	[214]
**324.**	Ganoderiol A triacetate	-	*G. sinense* (fruit bodies)	[274]
**325.**	Ganoderal A	ACE inhibitory activity (10^−5^ M)	*G* *. lucidum*	[277]
**326.**	Ganoderol A	ACE inhibitory activity (10^−5^M)	*G* *. lucidum*	[277]
**327.**	Lucidumol B	HIV-I Protease inhibitory activity (50 µM)	*G. lucidum* (spores)	[221]
**328.**	Ganodermanontiol	-	*G. lucidum* (spores)	[279]
**329.**	Ganodermanondiol	-	*G. lucidum* (fruit bodies)	[280]
**330.**	Ganoderic acid TR	Inhibitory effect on 5*α*-reductase (8.6 µM)	*G* *. lucidum*	[156]
**331.**	Ganodermic acid Ja	-	*G. lucidum* (mycelia)	[261]
**332.**	Ganodermic acid Jb	-	*G. lucidum* (mycelia)	[261]
**333.**	15*α*-Hydroxy-3-oxo-5*α*-lanosta-7,9,24(*E*)-triene-26-oic acid	Cytotoxicity against human HeLa cervical cancer cell lines (58 µM)	*G* *. lucidum*	[215]
**334.**	15*α*,26-Dihydroxy-5*α*-lanosta-7,9,24(*E*)-trien-3-one	Cytotoxicity against human HeLa cervical cancer cell lines (1 µM)	*G* *. lucidum*	[215]
**335.**	3*β*-Hydroxy-5*α*-lanosta-7,9,24(*E*)-trien-26-oic acid	Cytotoxicity against human HeLa cervical cancer cell lines (59 µM)	*G* *. lucidum*	[215]
**336.**	Epoxyganoderiol B	-	*G* *. lucidum*	[222]
**337.**	Epoxyganoderiol C	-	*G* *. lucidum*	[222]
**338.**	Ganoapplic acid F	Inhibitory effects for the proliferation of hepatic stellate cells (HSCs) induced through transforming growth factor-*β*1 (TGF-*β*1) in vitro	*G* *. applanatum*	[50,51]
**339.**	Ganoderic aldehyde TR	-	*G. lucidum*	[65]
**340.**	Ganoderic acid TR_1_	-	*G. lucidum*	[65]
**341.**	23-Hydroxy ganoderic acid S	-	*G. lucidum*	[65]
**342.**	Ganoellipsic acid A	-	*G. ellipsoideum*	[129]
**343.**	Ganoellipsic acid B	-	*G. ellipsoideum*	[129]
**344.**	Ganoellipsic acid C	-	*G. ellipsoideum*	[129]
**345.**	26-Methy-15*α*,22*β*-diacetoxy-7,9 (11),24-trien-26-oic ester	Moderate cytotoxic activity against the human cancer cell line NCI-H1650 (IC_50_ = 22.3 μM)	*G. capense*	[238]
**346.**	Methyl gibbosate L	Anti-adipogenesis activity—NE	*G. applanatum*	[41]
**347.**	Methyl ganoapplate F	Anti-adipogenesis activity—NE	*G. applanatum*	[41]
**348.**	Ganodeweberiol A	Cytotoxicity against HeLa cell line (IC_50_ = 31.6 μM)	*G. weberianum*	[77]
**349.**	Ganodeweberiol B	Significant α-glucosidase inhibitory activity	*G. weberianum*	[77]
**350.**	Ganodeweberiol C	Inhibits glucagon-inducedhepatic glucose production, inhibits hepatic glucose output through suppression hepatic cAMP accumulation, cytotoxicity against HeLa cell line (IC_50_ = 17.0 μM)	*G* *. weberianum*	[77]
**351.**	Ganodeweberiol D	-	*G* *. weberianum*	[77]
**352.**	Ganodeweberiol E	-	*G* *. weberianum*	[77]
**353.**	Ganodeweberiol F	Inhibits glucagon-inducedhepatic glucose production, inhibits hepatic glucose output through suppression hepatic cAMP accumulation	*G* *. weberianum*	[77]

Remark: NE = No Effect.

##### Triterpenoid Saponins

**Figure 11:**
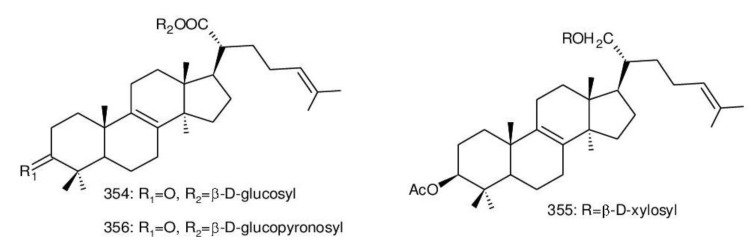
Chemical structures of several triterpenoid saponins (**354**–**356**).

The plant triterpenoids naturally exist in their glycosidic forms and are named as triterpenoid saponins [281]. More than 300 *Ganoderma* triterpenoids have already been found, although very few *Ganoderma* triterpenoid saponins have been identified [282]. *Ganoderma* triterpenoid saponins (Figure 11) are a kind of rare constituent, and until now only three triterpenoid saponins were isolated from *Ganoderma*. Furthermore, their glycosyl groups were located at C-21. The first triterpenoid saponin, 3*α*-acetoxy-5*α*-lanosta-8,24-dien-21-oic acid ester *β*-D-glucoside (**354**) from the fruiting bodies of *G. tsugae*, was reported in 1998. Its cytotoxicity analysis showed inhibition against Hep 3B cells through apoptosis [283]. Subsequently, Su et al. [206] isolated triterpenoid xyloside and tsugarioside B (**355**) from this fungus. Liu et al. [198] identified another triterpenoid saponin, named ganosinoside A (**356**), during studying on the chemical constituents from *G. sinense*.

Due to the rare nature of *Ganoderma* triterpenoid saponin, the modification (glycosylation) of natural *Ganoderma* triterpenoids into triterpenoid saponins is a promising strategy for both creating new compounds and expanding the bioactivities of *Ganoderma* triterpenoids.

#### 2.2.2. C29 Triterpenoids

*Ganoderma tropicum* has been widely used as a local remedy for coronary heart disease treatment, liver protection, and as a sleep aid [284]. The chemical investigation of its fruiting bodies led to a nortriterpenoid named 26-nor-11,23-dioxo-5*α*-lanost-8-en-3*β*,7*β*,15*α*,25-tetrol (**357**) [284]. The inhibitory activity against acetylcholinesterase (AChE) for compound **357** was tested. The results showed that **357** had low percentage inhibition (<10%) at the concentration of 100 μM, indicating no significant inhibitory activity against AChE [284]. Ganohainanic acid E (**358**) with 29-norlanostane skeleton was identified from *G. hainanense* for the first time. The evaluation of cytotoxicity showed that compound **358** had no inhibitory effect against HL-60, SMMC-7721, A-549, MCF-7, and SW480 cell lines [62]. Huang et al. [53] searched for active anticancer components in the fruiting bodies of *G. calidophilum* and isolated a previously undescribed lanostanoid species named as ganodecalone B (**359**). Phytochemical investigation of lanostane triterpenoids from *G. luteomarginatum* isolated two types of previously undescribed C29 structures named as (5*α*, 23*E*)-27-nor-lanosta-8,23-dien-3,7,25-trione (**360**) and (5*α*,23*E*)-27-nor-3*β*-hydroxylanosta-8,23-dien-7,25-dione (**361**) with an unusual 27-nor-lanostane carbon skeleton [285]. (5*α*,23*E*)-27-Nor-3*β*-hydroxylanosta-8,23-dien-7,25-dione (**361**) exhibited significant cytoxicity against HGC-27 cells (IC_50_ < 10 μM), but it’s cytotoxicity against LO2 cells was relatively low [285]. According to Su et al. [67], the investigation of *Ganoderma* triterpenoids with anti-inflammatory activities from *G. lucidum* extracted ganoluciduone B (**362**) as an unusual lanostane nortriterpenoid with 29 carbons. Ganoluciduone B (**362**) exhibited moderate inhibitory activity on nitric oxide production, with an inhibition rate of 45.5% at a concentration of 12.5 μM. Yang et al. [77] isolated a previously undescribed C29 compound ganodeweberiol H (363) from the fruiting bodies of *G. weberianum*, and which exhibited weak anti-inflammatory activity (IC_50_ = 40.71 μM) compared to the quercetin.

**Figure 12:**
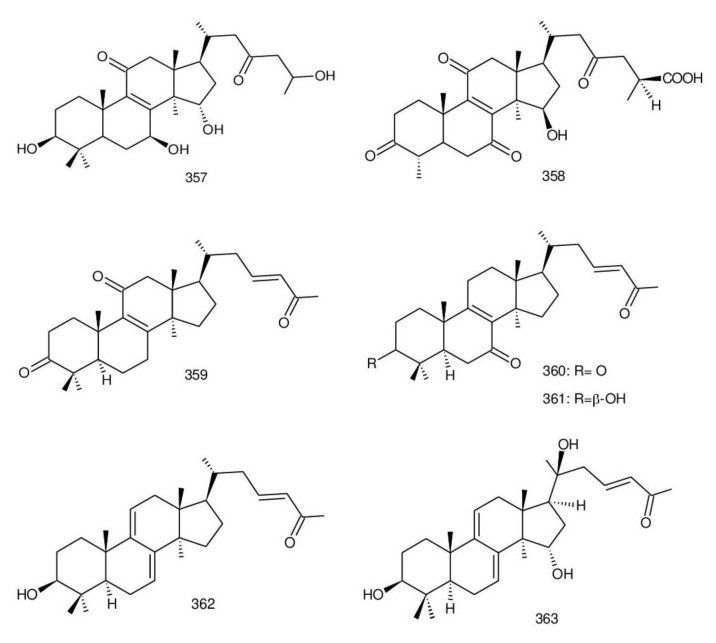
Chemical structures of several C29 triterpenoids (**357**–**363**).

Chemical structures of different C29 triterpenoids are shown in Figure 12.

#### 2.2.3. C27 Triterpenoids

Since Nishitoba et al. [286] first isolated C27 triterpenoids lucidenic acids A, B and C (**367**–**369**) from *G. lucidum* in 1984, a series of C27 triterpenoids were found one after another. Until now, several C27 triterpenoids have been isolated mainly from *G. lucidum* and *G. sinense*. Their structures also contain 8,9-ene or 7,9(11)-dien fractions, which are the same as those of C30 triterpenoids, except that C-25, C-26, and C-27 in the side chain were degraded as C27 triterpenoids [274,287,288]. Because the carboxyl and hydroxyl groups were present in C-24 and C-20, respectively, three C27 triterpenoids with a *γ*-lactone, named as ganolactone B (**392**), ganolactone A (**393**) and lucidenic lactone (**394**), were isolated [274,287,288].

According to Wei et al. [64], systemic investigation into the triterpenoids of *G. lucidum* isolated and identified three 27-nor Ganoderlactones structures with the C27 skeleton and were named as 7-oxo-ganoderlactone D (**403**), 21-hydroxyganoderlactone D (**404**), and ganoderlactone F (**405**). These three compounds displayed a moderate inhibitory effect on AChE. Ethyl lucidenate A (**368**) is a C27 triterpenoid separated from *G. lucidum* and displayed cytotoxic activity as previously reported [289]. In addition, ethyl lucidenate A has potent effects in reversing P-gp-mediated multidrug resistance. It may be a potential agent for reversing drug resistance in cancer chemotherapy [289]. The strange accumulation of melanin generates skin pigmentation and tyrosinase regulates melanin synthesis. Methyl lucidenate F (**365**), a C27 triterpenoid compound extracted from *G. lucidum* displayed a dose-dependent tyrosinase inhibitory activity, with an IC_50_ of 32.23 μM [65].

**Figure 13:**
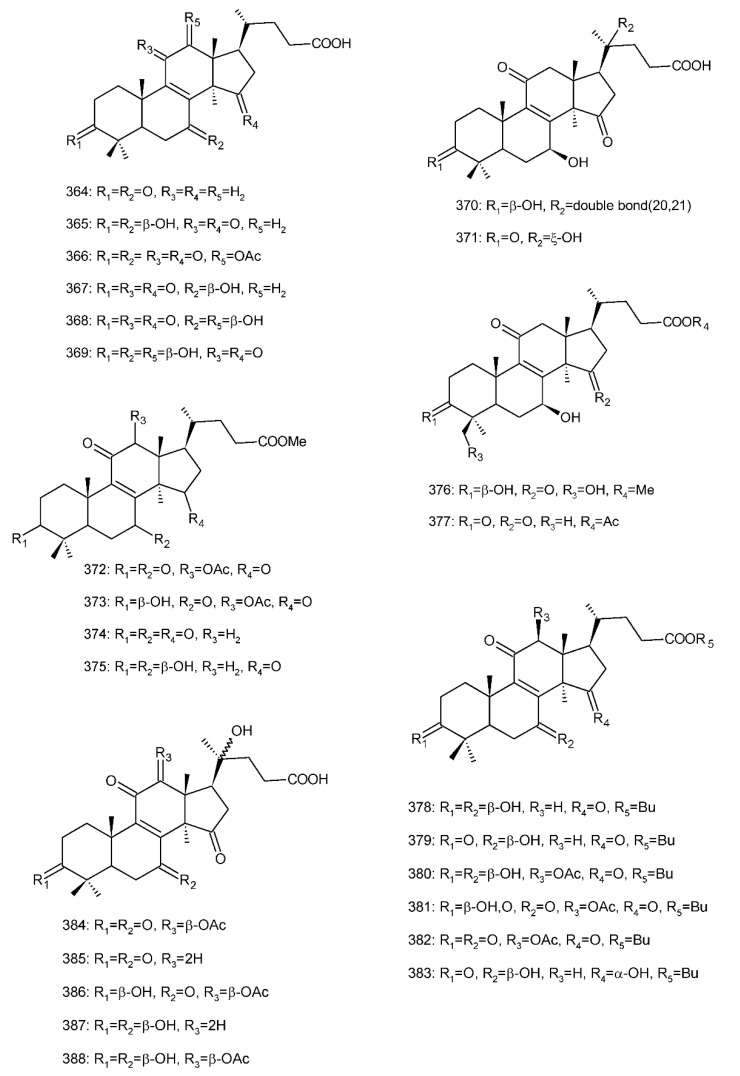

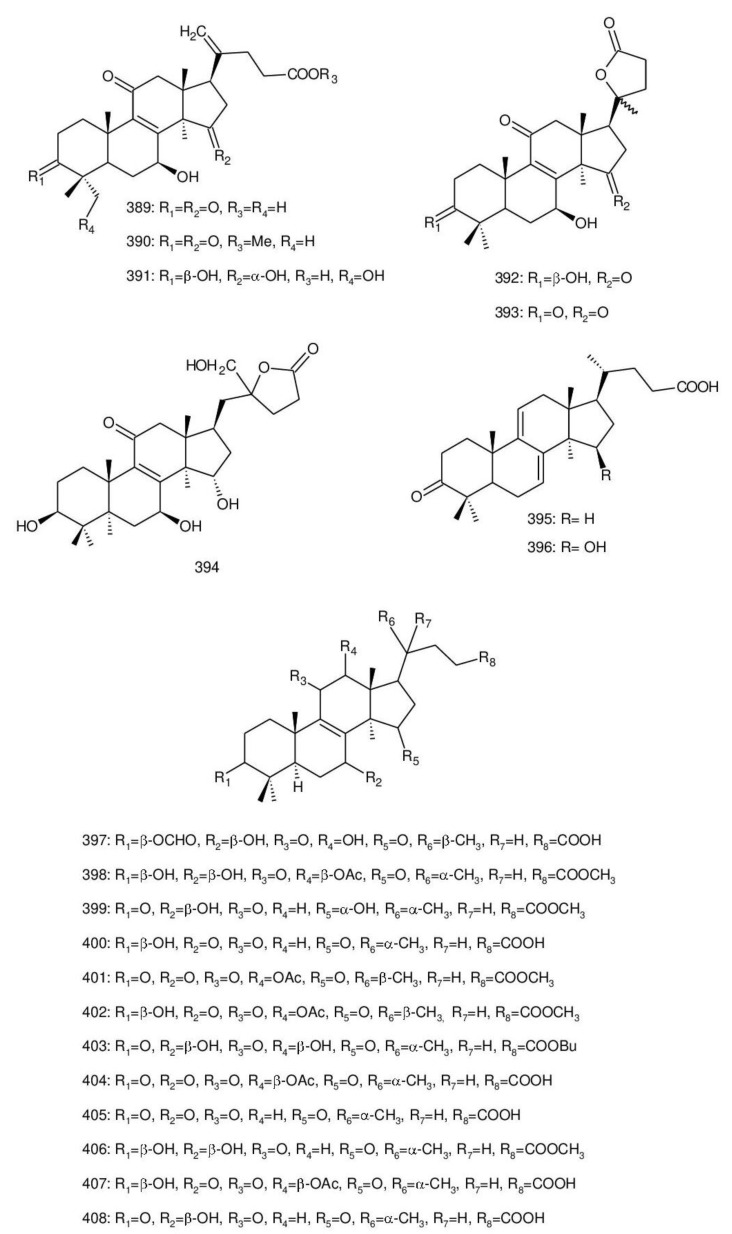

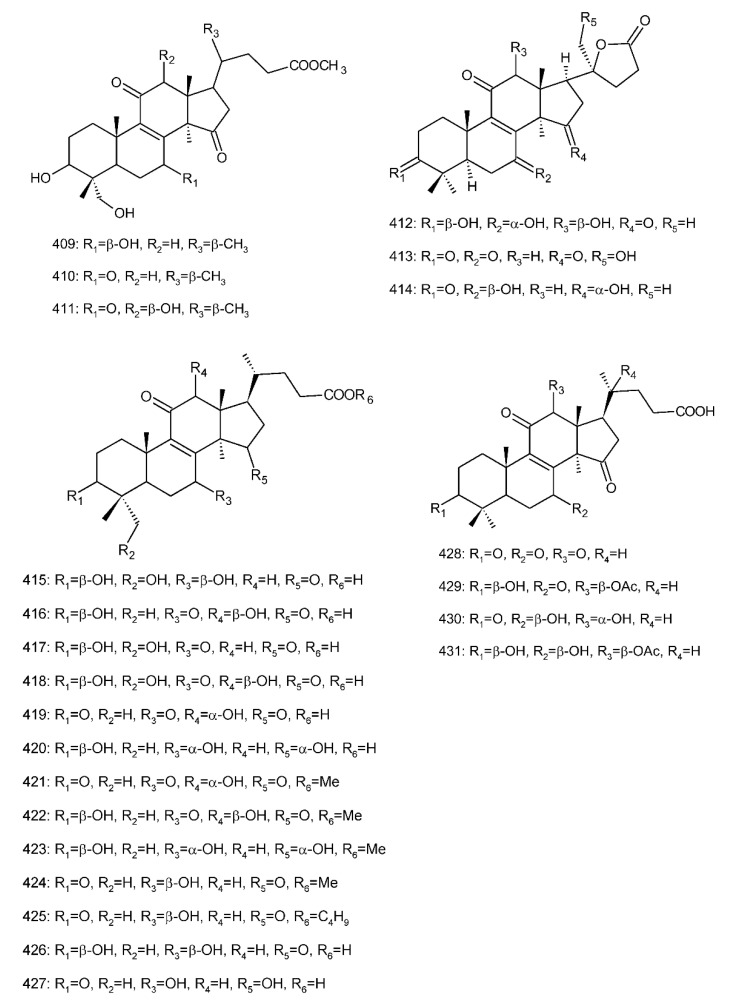
Chemical structures of several C27 triterpenoids (**364**–**431**).

Chemical structures of different C27 triterpenoids are shown in Figure 13 and their bioactivities are tabulated in Table 3.

#### 2.2.4. C25 Triterpenoids

**Figure 14:**
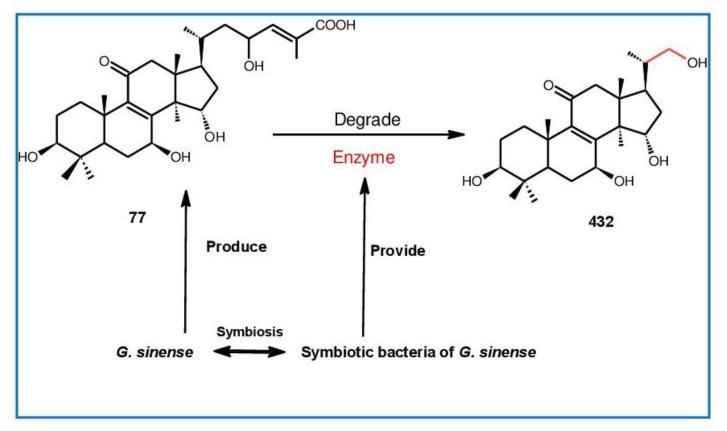
C25 Triterpenoids: the possible producing-pathway of **432** in *G. sinense*.

A pentanorlanostane, ganosineniol A (**432**), was isolated from the fruiting bodies of the macrofungus *G. sinense*. Compound **432** was the first pentanorlanostane triterpenoid from *Ganoderma*, while its analogues appear to be 23,24,25,26,27-pentanorlanost-8-en-3,22-diol [297], previously isolated from the bacteria of *Verticillium lecanii*, suggesting that ganosineniol A (**432**) would be produced or co-produced by the symbiotic bacteria of *G. sinense*. Liu et al. [198] also extracted ganolucidic acid γa (**77**) from this fungus, so they deduced that compound **77** was degraded into **432** by the related enzyme of symbiotic bacteria (Figure 14).

**Figure 15:**
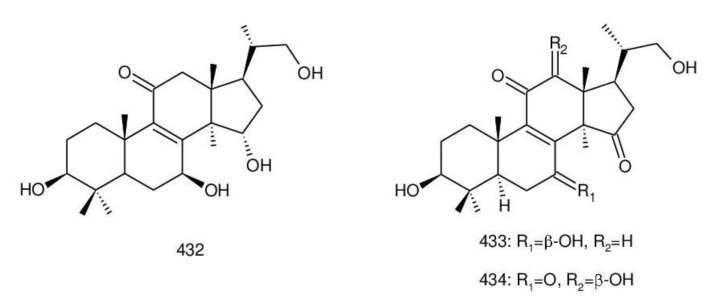
Chemical structures of several C25 triterpenoids (**432**–**434**).

Li et al. [66] isolated two new nortriterpenoids ganodrenol A (**433**), ganodrenol B (**434**) (Figure 15) from the ethanolic extracts of *G. lucidum*. The discovery of these compounds increased the chemical diversity of characteristic nortriterpenoids in *G. lucidum*. These nortriterpenoids displayed no significant cytotoxicity. In the Fatty Acid Amide Hydrolase (FAAH) inhibitory assay, the inhibitory rates of these compounds were below 30% [66].

#### 2.2.5. C24 Triterpenoids

**Figure 16:**
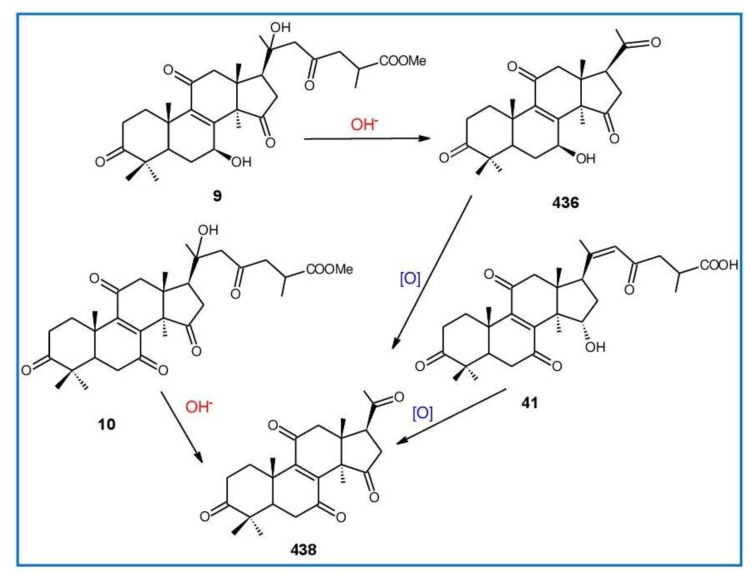
Retro-aldol condensations and oxidation of compounds **436** and **438**.

Nishitoba and the research group have studied the bitter triterpenoids from the fruiting bodies of *G. lucidum* since 1985. Besides C30 and C27 triterpenoids, C24 triterpenoids including lucidones A (**435**), B (**436**), C (**437**) and H (**438**) were also identified from this fungus [175,197,290,298], and they discussed how to produce these C24 triterpenoids. Nishitoba et al. [175] proposed that lucidones A (**435**), B (**436**) and C (**437**) might be artefacts and that lucidones B (**436**) and H (**438**) were derived from methyl ganoderate N (**9**) and methyl ganoderate O (**10**) under the alkaline conditions used during the isolation procedure. In order to confirm this deduction, they used 1 M KOH to treat compounds methyl ganoderate N and methyl ganoderate O (**9** and **10**) for 30 min to yield compounds lucidones B and H (**436** and **438**), respectively, which were determined using 1D NMR (Figure 16). Peng et al. [193] isolated a series of C24 triterpenoids, lucidones A (**435**), B (**436**), H (**438**) and D-G (**439**–**442**) from the MeOH extract of *G. resinaceum*, which was treated with satd aq. Na_2_CO_3_. This also indicated that these C24 triterpenoids were artefacts. Furthermore, lucidone H (**438**) was also formed by the PDC (pyridinium dichromate) oxidation of lucidone B (**436**) and ganoderenic acid G (**41**) following treatment with ozone followed by PDC [188].

Four new nortriterpenoid with A C24 skeleton named as lucidones I-L (**443**–**446**) were isolated and identified from the fruiting bodies of *G. resinaceum. α*-Glucosidase inhibitory activity was examined for each compound and lucidones K (**445**) and L (**446**) displayed no significant α-glucosidase inhibitory activity [299].

The 8*α*,9*α*-Epoxy-4,4,14*α*-trimethyl-3,7,11,15,20-pentaoxo-5*α*-pregnane (**447**) was isolated from the EtOAc extract of the fruiting bodies of *G. concinna* Ryv. (Ganodermataceae) was also a C24 triterpenoid [271]. Compared with the above lucidones, an 8,9-epoxy group was present in **447**. Furthermore, the evaluation of cytotoxicity showed that it induced apoptosis in human promyelocytic leukemia HL-60 cells [271]. In addition, Li et al. [66] isolated a new C24 natural compound ganodrenol C (**448**) from the ethanolic extracts of *G. lucidum*.

**Figure 17:**
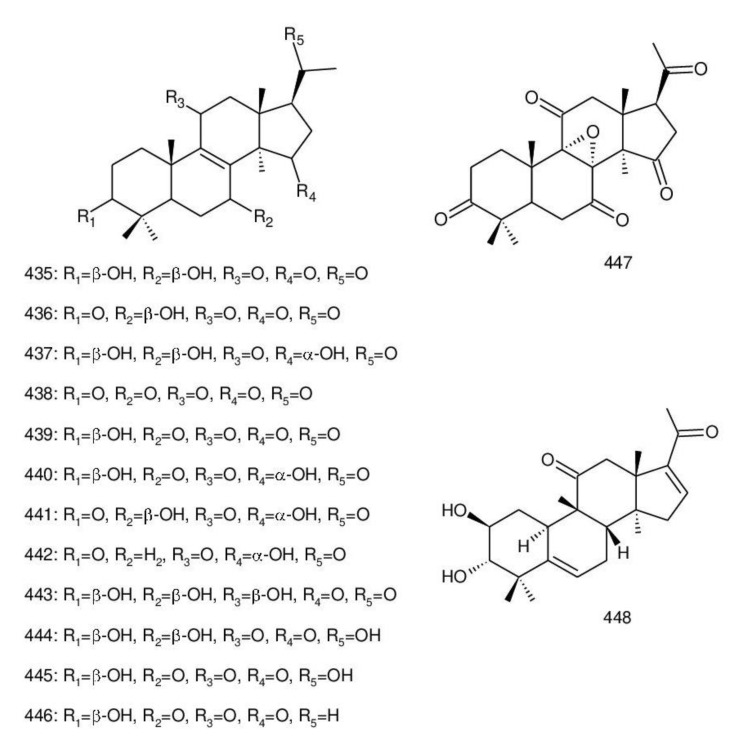
Chemical structures of several C24 triterpenoids (**435**–**448**).

Chemical structures of different C24 triterpenoids are shown in Figure 17**.**

#### 2.2.6. C31 Triterpenoids

Triterpenoids from *Ganoderma* were compared to the C30 skeleton and its degradation derivatives. However, two C31 triterpenoids, 3*α*-carboxyacetoxy-24-methylen-23-oxolanost-8-en-26-oic acid (**449**) and 3*α*-carboxyacetoxy-24-methyl-23-oxolanost-8-en-26-oic acid (**450**), were isolated from the fruiting bodies of *G. applanatum*, and were originally identified from the Basidiomycete fungi *Daedalea quercina* and *Daedaleopsis confragosa* var. *tricolor* [229]. Prior to the above investigation, Chairul et al. [300] isolated (**449**), (**450**) carboxyacetylquercinic acids and carboxyacetylquercinic acid derivative 2 (**451**) from *Ganoderma* spp. One year later, the other two new C31 triterpenoids, the 3*α*-acetoxy-16*α*-hydroxy-24-methylene-5*α*-lanost-8-en-21-oic acid (**452**) and the 3*a*-(3-hydroxy-5-methoxy-3-methyl-1,5-dioxopentyloxy)-24-methylene-5-lanost-8-en-21-oic acid (**453**), were obtained by Niu et al. [227] and compound **453** showed significant cytotoxic activity with an IC_50_ value of 2.5 μg/mL in the Hep-2 cell line, similar to that of the positive control, cisplatin (IC_50_ = 2.1 μg/mL) [227].

**Figure 18:**
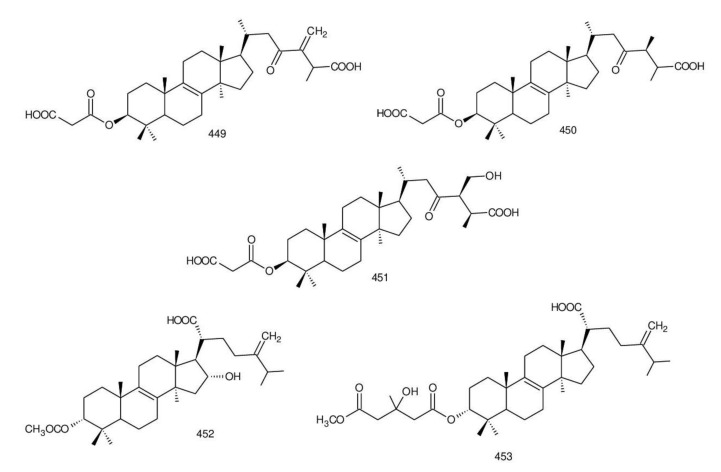
Chemical structures of several C31 triterpenoids (**449**–**453**).

Chemical structures of different C29 triterpenoids are shown in Figure 18.

#### 2.2.7. Rearranged Novel Triterpenoids

Liu et al. [135] summarized the potential triterpenoids biosynthesis pathway in *G. lucidum* and found that the pathway contained 14 steps catalyzed by different enzymes. The first 11 steps are the common steps for terpenoid skeleton biosynthesis, and the last three steps may be specific for different triterpenes in different species. It has been reported that this specific modification (cyclization, oxidation, hydroxylation and glycosylation) is carried out by cytochrome P450, glycosyltransferases and other enzymes [301,302,303,304,305,306]. Therefore, the plentiful enzymes or enzyme systems of *Ganoderma* produced diverse triterpenoids and different species may have unique novel structures.

**Figure 19:**
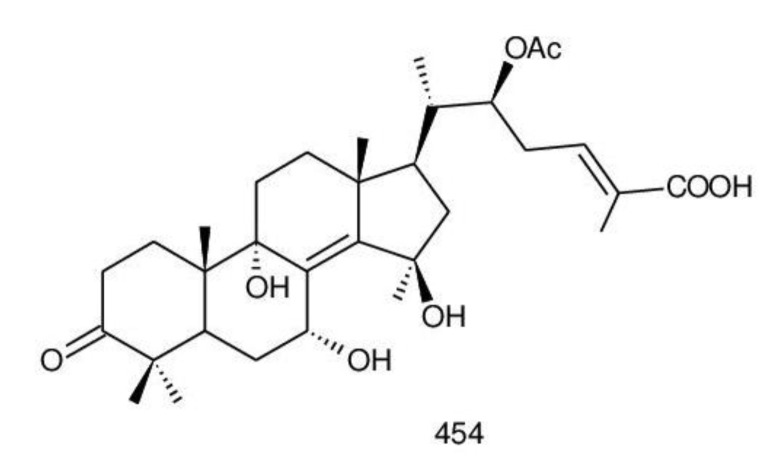
Chemical structure of ganorbiformin A (**454**) as a rearranged novel triterpenoid.

**Figure 20:**
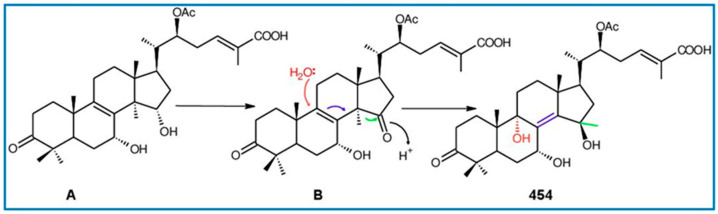
A possible mechanism to account for the formation of **454**.

Ganorbiformin A (**454**) (Figure 19) is an unusually rearranged analogue and was isolated from the cultures of *G. orbiforme* BBC 22324 [36]. The former researchers proposed a possible mechanism to account for the formation of **454** (Figure 20). This compound was the C-15 alcohol oxidation of **A** to the ketone **B**, whose methyl group (Me-30) on the *α*-face migrated to the neighboring ketone carbon (C-15) under acid catalysis [36].

**Figure 21:**
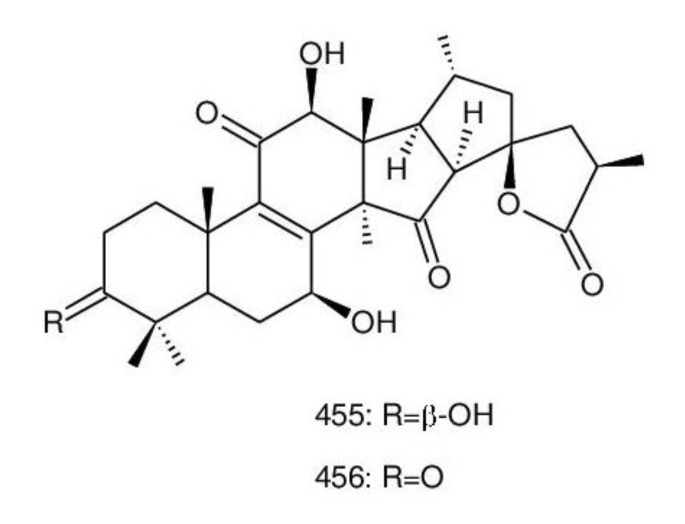
Chemical structures of ganosporelactones B (**455**) and A (**456**) as rearranged novel triterpenoids.

Ganosporelactones B (**455**) and A (**456**) (Figure 21) are two novel pentacyclic triterpenoids, which were isolated from the spores of *G. lucidum* [307]. They may be biogenetically derived from the lanostane skeleton through the construction of the C-16 and C-23 bonds [307].

**Figure 22:**
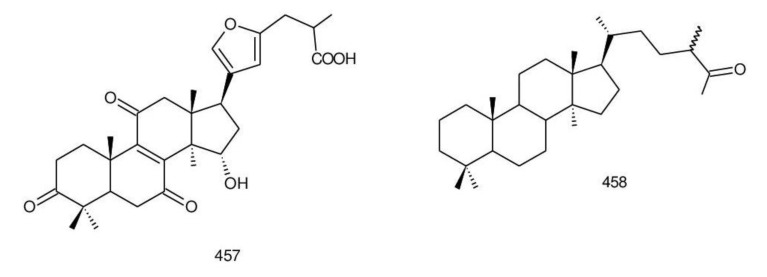
Chemical structures of furanoganoderic acid (**457**) and 24*ζ*-methyl-5*α*-lanosta-25-one (**458**) as rearranged novel triterpenoids.

Furanoganoderic acid (**457**) (Figure 22), a lanostane triterpenoid with a furan ring (21,23-epoxy) in the side chain, was isolated from the fruiting bodies of *G. applanatum* [188]. The further study on the chemical constituents of this macrofungus led to the isolation of 24*ζ*-methyl-5*α*-lanosta-25-one (**458**) (Figure 22) [308]. The analysis of its structure showed that it was a lanostane triterpenoid without any substituent, except for a keto group on the side chain, and the 2D NMR determined that Me-26 had shifted to C-24 [308].

**Figure 23:**
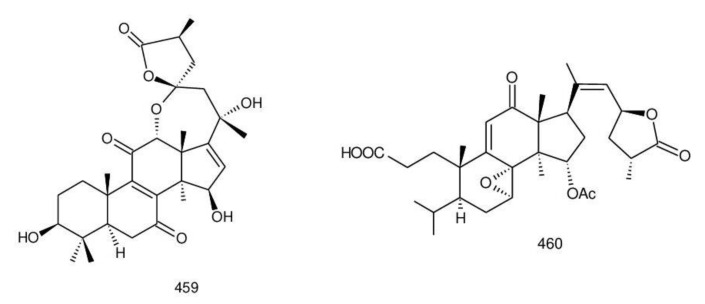
Chemical structures of polyoxygenated lanostane triterpenoids: austrolactone (**459**) and australic acid (**460**).

Two polyoxygenated lanostane triterpenoids, austrolactone (**459**) and australic acid (**460**) (Figure 23), were extracted from *G. australe* [276]. The austrolactone (**459**) was a C30 triterpenoid spirolactone, and was formed through the ketalation of the O-atoms (C-12 and COOH-26) and the carbonyl group (C-23). The australic acid (**460**) was 3,4-*seco*-8,9-epoxy-23→26 lactone and was similar to elfvingic acid H isolated from *Elfvingia applanata* (Pers.) Pat. In the cytotoxicity assay, compounds **459** and **460** (IC_50_ = 94 µM) specifically inhibited the viability and growth of the HL-60 cells through activation of the apoptotic cell-death pathway as demonstrated by morphological and biochemical analyses [276].

**Figure 24:**
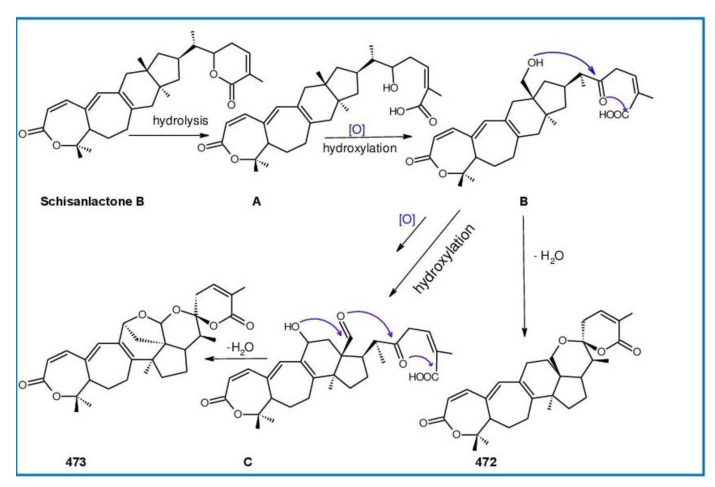
Possible biogenetic pathways for triterpenoid lactones ganodermalactones G (**472**) and F (**473**).

A number of triterpenoid lactones (**461**–**471**) have been found from the rare species *G. colossum*. These types of triterpenoids have also been reported in plants including schisanlactones A and B (*Schiandra* sp.) [309], kadsulactone A *(Kadsura heteroclita*) [310] and lancilactones A-C (*K. lancilimba*) [311]. Their structures were characterized by: (1) ring A: 3,4-*seco* or 3,4-*seco*-3→4 lactone (seven-membered lactone); (2) ring B: 9,19-cyclic-9,10-*seco* (seven-membered ring) or six-membered ring; (3) side chain: 24-ene-22→26 lactone (*α*,*β*-unsaturated-*δ*-lactone) or 22-hydroxy-24-ene-26-acid. Except for the above structures, it is noted that two triterpenoid lactones, ganodermalactones G and F (**472** and **473**) containing a spiroketal-lactone and bicyclo-spiroketal-lactone skeletons, were first isolated from the EtOAc extract of the cultured biomass of *Ganoderma sp*. KM01 and a possible biogenetic pathway for ganodermalactone G (**472**) and ganodermalactone F (**473**) were proposed (Figure 24) [210].

Further bioactivity assay showed: (1) Colossolactone V (**461**), colossolactone VI (**462**) and colossolactone E (**467**) exhibited inhibitory activity against HIV-1 protease with IC_50_ values of 9.0, >100 and 8.0 μg/mL, respectively, El Dine 2008b [312]. (2) Colossolactone C (**464**), colossolactones D (**466**), E (**467**), F (**468**) and colossolactone G (**471**) showed moderate cytotoxicity against L-929, K-562 and HeLa cells with IC_50_ values ranging from 15 to 35 μg/mL. However, they showed no antimicrobial activity against a spectrum of bacteria and fungi [207]. (3) Colossolactone H (**470**) as a new triterpene dilactone produces more cytotoxic than colossolactone G (**471**) and shows cytotoxicity against lung, breast, and colon cancers and hepatoma cells, suggesting that it may be a beneficial adjuvant for the therapy involved in treating a wide range of cancers [59]. Compounds **468** and 23-hydroxycolossolactone E (**469**) were active against *P. syringae* and *B. subtilis* [211]. Ganodermalactone F (**473**) [313]) and colossolactone E (**467**) showed antimalarial activity against *Plasmodium falciparum* with IC_50_ values of 10.0 and 10.0 μM, respectively [210].

**Figure 25:**
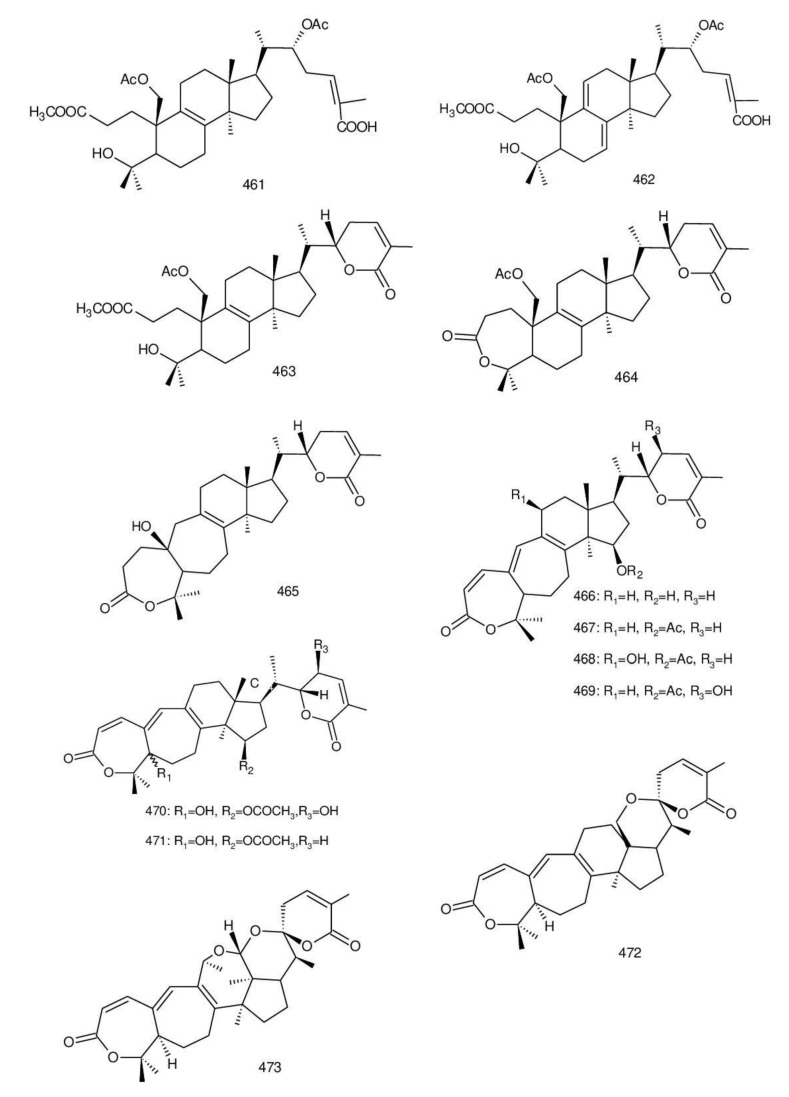
Chemical structures of several rearranged novel triterpenoids (**461**–**473**).

Chemical structures of different rearranged novel triterpenoids are shown in Figure 25.

**Figure 26:**
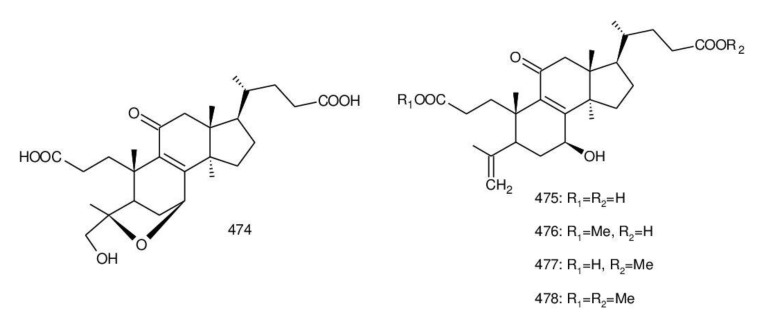
Chemical structures of several rearranged novel triterpenoids (**474**–**478**).

Fornicatins A (**474**), B (**475**) and D-F (**476**–**478**) (Figure 26) were 3,4-*seco*-trinortriterpenoids. Among them, fornicatins A and B (**474** and **475**) were first isolated from the fruiting bodies of *G. fornicatum* and tested for their inhibitory effects on rabbit platelet aggregation induced through either a platelet activating factor (PAF), adenosine diphos-phate (ADP), or arachidonic acid (AA) [314]. These compounds were also obtained from *G. cochlear*. Fornicatins A (**474**), D (**476**) and F (**478**) displayed in vitro hepatoprotective activities by lowering the ALT and AST levels in HepG2 cells treated with H_2_O_2_ [57].

**Figure 27:**
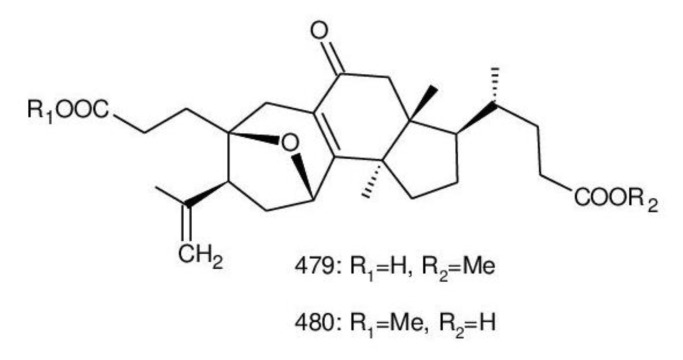
Chemical structures of cochlate A (**479**) and cochlate B (**480**) as rearranged novel triterpenoids.

**Figure 28:**
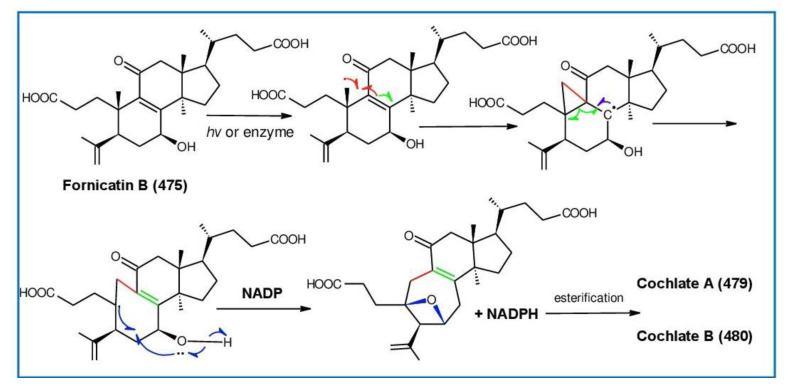
The biosynthetic route of cochlates A (**479**) and B (**480**).

Cochlates A and B (**479** and **480**) (Figure 27) with a 3,4-s*eco*-9,10-*seco*-9,19-cyclo skeleton were a pair of isomers and were isolated from *G. cochlear* [57]. The only difference between **479** and **480** was the position of the ester. Considering the wide distribution of fornicatin B (**475**) in *G. cochlear*, cochlates A and B (**479** and **480**) could be derived from a modification of **475**. Therefore, a possible biosynthetic route of **479** and **480** was proposed, as shown in Figure 28 [57].

**Figure 29:**
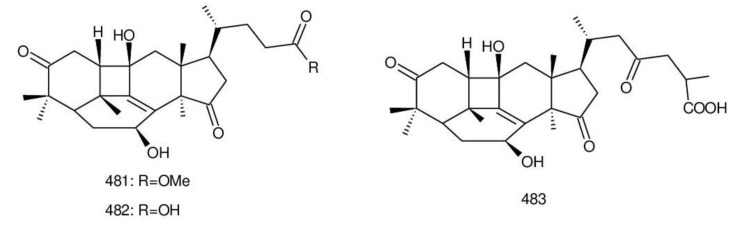
Chemical structures of several rearranged novel triterpenoids (**481**–**483**).

**Figure 30:**
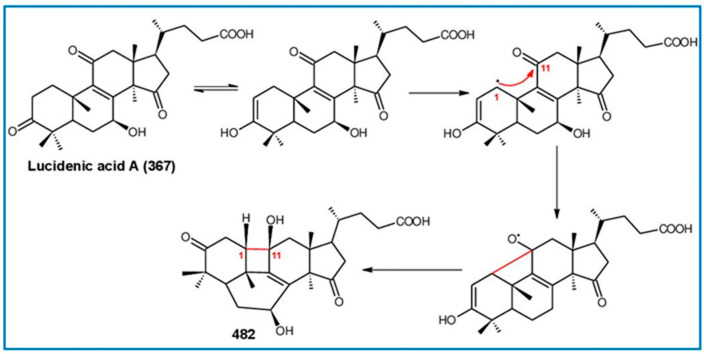
The plausible biosynthesis pathway of **482**.

According to the literature, the unprecedented four-membered framework of methyl ganosinensate A (**481**), ganosinensic acid A (**482**) and ganosinensic acid B (**483**) (Figure 29) was produced by a bond across C-1 to C-11 due to the radical reaction (Figure 30) [315]. These three new triterpenoids were isolated from *G. sinense* and showed weak cytotoxicity against HL-60, SMMC-7721, A-549, MCF-7 and SW480 cell lines [315].

**Figure 31:**
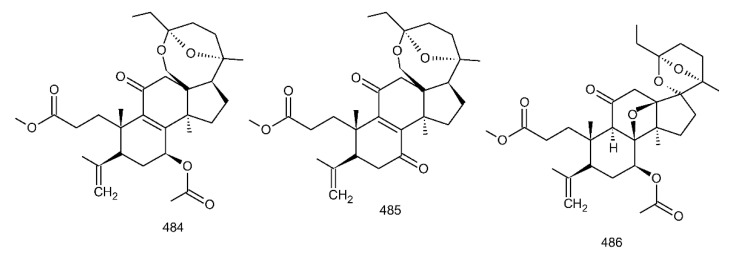
Chemical structures of several rearranged novel triterpenoids (**484**–**486**).

Three new nortriterpenes, ganoboninketals A-C (**484**–**486**) (Figure 31) featuring rearranged 3,4-*seco*-27-norlanostane skeletons and complex polycyclic systems were isolated from *G. boninense* [52]. Compounds **484**–**486** showed anti-plasmodial activity against *Plasmodium falciparum* with IC_50_ values of 4.0, 7.9, and 1.7 μM, respectively, and presented NO inhibition in the LPS-induced macrophages with IC_50_ values in the range of 24–100 μM. Furthermore, compounds **484** and **485** displayed weak cytotoxicity against A549 cells. Compound **486** showed weak cytotoxicity toward HeLa cells [52].

**Figure 32:**
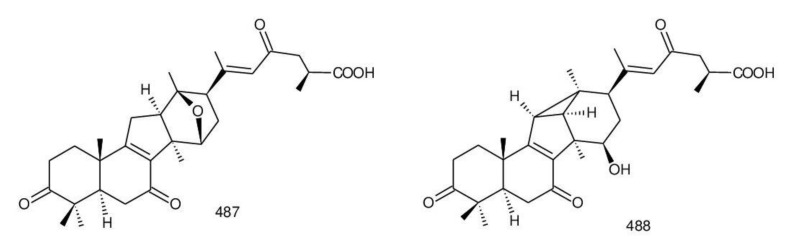
Chemical structures of ganoapplanic acid A (**487**) and ganoapplanic acid B (**488**) a**s** rearranged novel triterpenoids.

Ganoapplanic acids A (**487**) and B (**488**) (Figure 32) are two rearranged lanotane-type triterpenoids isolated from *G. applanatum* with a 6/6/5/6-fused tetracyclic skeleton. Ganoapplanic acid B (**488**) represents the first example of a rearranged triterpenoid with a three-membered carbon ring and the compound (**487**) exhibited inhibitory effects for the proliferation of hepatic stellate cells (HSCs) induced by transforming growth factor-β1 (TGF-β1) in vitro [50].

**Figure 33:**
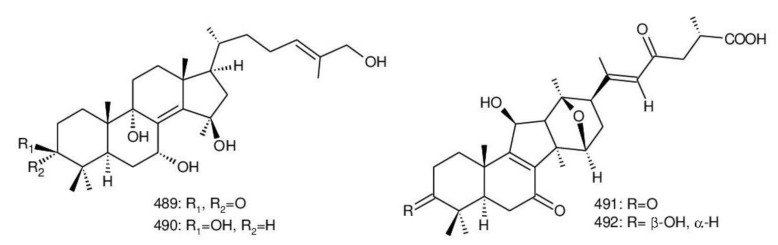
Chemical structures of several rearranged novel triterpenoids (**489**–**492**).

Isaka et al. [234] isolated two new rearranged lanostanes, ganocasurarins A (**489**) and B (**490**) (Figure 33), from *G. casuarinicola*, sharing the same carbon skeleton with only ganorbiformin A (**454**). Ganodapplanoic acids A (**491**) and B (**492**) (Figure 33) are another two rearranged 6/6/5/6-fused lanostane-type triterpenoids in *G. applanatum* with an unusual C-13/C-15 oxygen bridge moiety. But these two compounds did not effectively repress adipogenesis [316].

**Figure 34:**
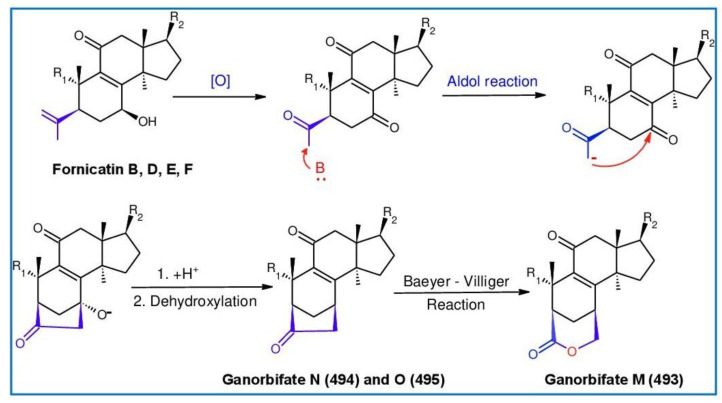
Plausible biosynthetic pathway of ganorbifates M, N, and O (**493**–**495**).

The chemical investigation of *G. cochlear* isolated a novel type of rearranged compound named as ganorbifate M (**493**), and compounds ganorbifate N (**494**) and ganorbifate O (**495**) showed a carbon skeleton of 3,4-*seco*-25,26,27,28-tetranorlanostane triterpenoid [58]. A plausible biosynthetic pathway of compound ganorbifates M, N and O (**493**–**495**) was proposed by using the Aldol reaction and Baeyer–Villiger reaction during the study (Figure 34) [58].

## 3. Conclusions

This review concludes that *Ganoderma* contains various pharmacologically active triterpenoids and that GTs are one of the main described metabolic constituents with different structural types, which are related to the diverse range of biological activities. According to the GTs reported from 1984 to 2022, we found that 8,9-ene ganoderma acid and 7,9(11)-diene ganoderma alcohol showed significant anti-HIV-1, anti-HIV-1 protease and cytotoxic activities, laying a foundation for further investigations of bioactive analyses. The main ganoderma acid and ganoderma alcohol can be isolated from *Ganoderma*, which suggested that GTs can be used for the control of various diseases. In addition, different species of *Ganoderma* possessed some specific lanostane triterpenoid, and some novel skeletons only appeared in specific species. Meanwhile, the diversity of *Ganoderma* species mainly reveals morphological differences, which were related to the geographic position and biotic environment. However, these conditions led to the production of different types of GTs. In other words, the types of GTs may influence the morphology of *Ganoderma*. Furthermore, the biosynthetic pathway of GTs has been proven at the gene level. Thus, the relationship of the types of GTs and morphology could be verified through further gene analyses [142,317,318,319,320].

*Ganoderma* triterpenoids are structurally and stereochemically complex small molecules. In addition, each of these compounds possesses sites of diversification, allowing for the facile and rapid creation of dozens of complex compounds for drug screening. In recent years, researchers have focused on GTs and their broad spectrum of bioactivities. Natural products are typically complex molecules that have enjoyed notable success in drug discovery [321,322,323,324]. Here, the crucial tasks include the completion of clinical trials and the elaboration of high-quality *Ganoderma* spp. derived products with sustainable production via standard procedures.

Currently, the *Ganoderma* industry earns billions of dollars via cultivation or wild collections, consumption as tea or alcoholic beverages and the use of nutraceuticals, with the industry offering thousands of products to the markets [325,326,327]. With great potential for the *Ganoderma* market and industry, it is becoming increasingly essential that the active ingredients such as GTs are identified. This will help enhance existing market trends and future *Ganoderma* industry growth in terms of both value and volume. Although *Ganoderma* triterpenoids are found to be one of the main bioactive constituents, more attention should be paid in future studies in order to detect other bioactive compounds.

## Figures and Tables

**Table 3:** C27 Triterpenoids and bioactivities from *Ganoderma*.

No.	Trivial Names	Bioactivities (IC_50_/MIC or ED_50_)	Sources *Ganoderma* Species	References
**364.**	4,4,14*α*-Trimethyl-3,7-dioxo-5*α*-chol-8-en-24-oic acid	Moderate cytotoxicity against HeLa cervical cells (48.0 μM)	*G. lucidum*	[215]
**365.**	Lucidenic acid N	Cytotoxicity against HL-60 (64.5 μM), HepG2 (2.06 × 10^−4^ μM), HepG 2,2,5 (1.66 × 10^−3^ μM), p388 cells (1.20 × 10^−2^ μM)	*G. lucidum*	[47,180]
**366.**	Lucidenic acid D	-	*G. lucidum*	[290]
**367.**	Lucidenic acid A	Cytotoxicity against HL-60 (61.0 μM), HepG2 (1.64 × 10^−4^ μM), HepG2,2,15 (2.05 × 10^−4^ μM), κB (16.9 μM), CCM2 (27.15 μM), p388 cells (1.70 × 10^−2^ μM)	*G. lucidum*	[47,127, 180,286]
**368.**	Lucidenic acid B	Cytotoxicity against HL-601 (9.3 μM), reduces PMA- induced MMP-9 activity and anti-invasive effects	*G. lucidum*	[47,286, 291]
**369.**	Lucidenic acid C	Cytotoxicity against HL-60 (45.2 μM)	*G. lucidum*	[47,286, 291]
**370.**	20(21)-Dehydrolucidenic acid N	Anti-HIV protease (48.0 μM)	*G. sinense*	[192]
**371.**	20-Hydroxylucidenic acid A	Anti-HIV protease—NE	*G. sinense*	[192]
**372.**	Methyl lucidenate D	-	*G. lucidum*	[184]
**373.**	Methyl lucidenate E	-	*G. lucidum*	[184]
**374.**	Methyl lucidenate F	-	*G. lucidum*	[184]
**375.**	Methyl lucidenate N	Cytotoxicity (inhibition of triglyceride accumulation in between 45%–50% at 80 μM during the differentiation of 3T3-L1 preadipocytes)	*G. lucidum*	[292]
**376.**	Methyl lucidenate Ha	-	*G. sinense*	[198]
**377.**	Ethyl lucidenate A	Cytotoxicity against HL-60 (25.9 μM/mL), CA46 cells (20.4 μM/mL)	*G. lucidum*	[242]
**378.**	Butyl lucidenate N	Inhibits adipocyte differentiation in 3T3-L1 cells: inhibition of lipid droplet formation (56% at 40 μg/mL)	*G. lucidum*	[183]
**379.**	Butyl lucidenate A	Inhibits adipocyte differentiation in 3T3-L1 cells: inhibition of lipid droplet formation (46%–48% at 40 μg/mL)	*G. lucidum*	[183]
**380.**	Butyl lucidenate P	Anti-inflammation (7.4 μM)	*G. lucidum*	[293]
**381.**	Butyl lucidenate D_2_	Anti-inflammation (35 μM)	*G. lucidum*	[293]
**382.**	Butyl lucidenate E_2_	Anti-inflammation (6.4 μM)	*G. lucidum*	[293]
**383.**	Butyl lucidenate Q	Anti-inflammation (4.3 μM)	*G. lucidum*	[293]
**384.**	20-Hydroxylucidenic acid D_2_	-	*G. lucidum*	[294]
**385.**	20-Hydroxylucidenic acid F	-	*G. lucidum*	[294]
**386.**	20-Hydroxylucidenic acid E_2_	-	*G. lucidum*	[294]
**387.**	20-Hydroxylucidenic acid N	-	*G. lucidum*	[294]
**388.**	20-Hydroxylucidenic acid P	-	*G. lucidum*	[294]
**389.**	20(21)-Dehydrolucidenic acid A	-	*G. lucidum*	[294]
**390.**	Methyl 20(21)-dehydrolucidenate A	-	*G. lucidum*	[294]
**391.**	Lucidenic acid O	Inhibition (DNA polymerase *α* and rat DNA polymerase *β*), anti-HIV-1	*G. lucidum*	[288]
**392.**	Ganolactone B	-	*G. sinense*	[274]
**393.**	Ganolactone A	Anti- inflammation—NE	*G. lucidum*	[295]
**394.**	Lucidenic lactone	Inhibition (DNA polymerase *α* and rat DNA polymerase *β*), anti-HIV-1	*G. lucidum*	[288]
**395.**	4,4,14-Trimethyl-5*α*-chol-7,9(11)-dien-3-oxo-24-oic Acid	Brain-derived neurotrophic factor-like neuronal survival-promoting activity	*G. lucidum*	[296]
**396.**	Ganoderic acid Jd	Cytotoxicity—NE	*G. sinense*	[198]
**397.**	3*β*-Oxo-formyl-7*β*,12*β*-dihydroxy-4,4,14*α*-trimethyl-5*α*-chol-11,15- dioxo-8-en(*E*)-24-oic acid	-	*G* *. lucidum*	[213]
**398.**	Methyl lucidenate P	-	*G. lucidum* (fruit bodies)	[185]
**399.**	Methyl lucidenate Q	-	*G. lucidum* (fruit bodies)	[185]
**400.**	3*β*-Hydroxy-4,4,14-trimethyl-7,11,15-trioxochol-8-en-24-oic acid	Cytotoxicity against p388 cell (18.00 μM), HeLa cell (12.70 μM), BEL-7402 cell (22.00 μM), SGC-7901 cell (1.50 μM)	*G. lucidum* (fruit bodies)	[216]
**401.**	Methyl lucidenate D_2_	-	*G. lucidum* (fruit bodies)	[217]
**402.**	Methyl lucidenate E_2_	-	*G. lucidum* (fruit bodies)	[217]
**403.**	t-Butyl lucidenate B	Inhibitory effect on adipocyte differentiation in 3T3-L1 cells	*G. lucidum* (fruit bodies)	[292]
**404.**	Lucidenic acid D_2_	-	*G. lucidum* (fruit bodies)	[185]
**405.**	Lucidenic acid F	-	*G. lucidum* (fruit bodies)	[212]
**406.**	Methyl lucidenate C	Cytotoxicity against human HeLa cervical cancer cell lines (101 uM)	*G* *. lucidum*	[215]
**407.**	Lucidenic acid E_2_	-	*G. lucidum* (fruit bodies)	[185]
**408.**	Lucideric acid A	Cytotoxicity against human HeLa cervical cancer cell lines—NE	*G* *. lucidum*	[215]
**409.**	Methyl lucidenate H	-	*G. lucidum* (fruit bodies)	[175]
**410.**	Methyl lucidenate I	-	*G. lucidum* (fruit bodies)	[175]
**411.**	Methyl lucidenate J	-	*G. lucidum* (fruit bodies)	[175]
**412.**	7-Oxo-ganoderlactone D	AChE inhibitory effect (91.2 μM)	*G. lucidum*	[64]
**413.**	21-Hydroxyganoderlactone D	AChE inhibitory effect (177.0 μM)	*G* *. lucidum*	[64]
**414.**	Ganoderlactone F	AChE inhibitory effect—NE	*G. lucidum*	[64]
**415.**	Lucidenic acid H	-	*G. lucidum* (fruit bodies)	[65]
**416.**	Lucidenic acid L	-	*G. lucidum* (fruit bodies)	[65]
**417.**	Lucidenic acid I	-	*G. lucidum* (fruit bodies)	[65]
**418.**	Lucidenic acid J	-	*G. lucidum* (fruit bodies)	[65]
**419.**	Lucidenic acid K	-	*G. lucidum* (fruit bodies)	[65]
**420.**	Lucidenic acid M	-	*G. lucidum* (fruit bodies)	[65]
**421.**	Methyl lucidenate K	-	*G. lucidum* (fruit bodies)	[65]
**422.**	Methyl lucidenate L	-	*G. lucidum* (fruit bodies)	[65]
**423.**	Methyl lucidenate M	-	*G. lucidum* (fruit bodies)	[65]
**424.**	Methyl lucidenate A	-	*G. lucidum* (mycelia)	[65]
**425.**	n-Butyl lucidenate A	-	*G. lucidum* (fruit bodies)	[65]
**426.**	n-Butyl lucidenate N	-	*G. lucidum* (fruit bodies)	[65]
**427.**	7,15-Dihydroxy-4,4,14-trimethyl-3,11-dioxochol-8-en–24-oic acid	-	*G. lucidum* (fruit bodies)	[65]
**428.**	Lucidenic acid D_1_	-	*G. lucidum* (fruit bodies)	[65]
**429.**	Lucidenic acid E	-	*G. lucidum* (fruit bodies)	[65]
**430.**	Lucidenic acid E_1_	-	*G. lucidum* (fruit bodies)	[65]
**431.**	Lucidenic acid P	-	*G. lucidum* (fruit bodies)	[65]

Remark: NE = No Effect.

## Data Availability

Not applicable.

## Abbreviations

The abbreviations including in the text are reported alphabetically.

AChE	Acetylcholinesterase
ALT	Alanine aminotransferase
AST	Aspartate aminotransferase
EV71	Enterovirus 71
FAAH	Fatty Acid Amide Hydrolase
FPPs	Farnesyl diphosphate synthase
GA	Ganoderic Acid
GAS	Ganodermic acid S
GTs	*Ganoderma* Triterpenoids
HSCs	Hepatic stellate cells
HMGR	3-Hydroxy-3-methylglutaryl-CoA reductase
HMGS	3-Hydroxy-3-methylglutaryl-CoA synthase
IC_50_	Half-maximal inhibitory concentration
ID_50_	Inhibitory dose-50
LS	Lanosterol synthase
MIC	Minimum Inhibitory Concentration
MVA	Mevalonate pathway
MVD	Phosphomevalonate decarboxylase
NOESY	Nuclear Overhauser Effect Spectroscopy
OSC	2,3-Oxidosqualene lanosterol cyclase
PDC	Pyridinium dichromate
SA	Salicylic Acid
SQS	Squalene synthase
TPA	12-O-Tetradecanoylphorbol 13-acetate
TGF-β1	Transforming growth factor-β1
uPA	Urokinase-plasminogen activator
